# Bathochromic Shift of Fluorescence Peak in Dipyrrolo[1,2-*a*:2′,1′-*c*]quinoxaline by Introducing Each of Electron-Donating and Electron-Withdrawing Substituent

**DOI:** 10.3390/molecules28072896

**Published:** 2023-03-23

**Authors:** Shoji Matsumoto, Makoto Takamori, Motohiro Akazome

**Affiliations:** Department of Applied Chemistry and Biotechnology, Graduate School of Engineering, Chiba University, 1-33 Yayoi-cho, Inage-ku, Chiba 263-8522, Japan

**Keywords:** dipyrroloquinoxaline, fluorescence, bathochromic shift, substituent effect, energy gap

## Abstract

Development of organic fluorophore is an important theme. Especially, the fluorophores with longer fluorescence peaks are useful to biological probes. One of the methods to change the fluorescence peak is the introduction of substituents. However, opposing characteristics of the substituents lead to different changes in the fluorescence peaks. Furthermore, the introduction of the substituent also affects their electric properties. Thus, if the materials were developed with the substituent effect on the optical and electric properties separately, it will be useful to design the functional materials related to both optical and electric properties. Herein, we investigated the substituent effect of dipyrrolo[1,2-*a*:2′,1′-*c*]quinoxalines on fluorescence properties. We synthesized the compounds bearing electron-donating or electron-withdrawing substituents at the benzene ring on dipyrrolo[1,2-*a*:2′,1′-*c*]quinoxaline, which would have more direct influence on the optical properties. By introducing each substituent at the 6 position of dipyrrolo[1,2-*a*:2′,1′-*c*]quinoxaline, the bathochromic shift was observed in the fluorescence spectra. In the case of fluorine substituent, the change of the fluorescence peak reached was about 19 nm. Using a TDDFT calculation, we explained the reason for such a substituent effect that large on the increment of LUMO energy or decrement of HOMO energy occurred by introducing electron-withdrawing or electron-donating substituents at the 6 position, respectively. The substituent effect on the change of orbital energies is typical although the different characteristics of substituents resulted in the similar tendency about the change of fluorescence peak. Furthermore, with the introduction of phenyl substituents at the 3 and 10 positions, we achieved 40–50 nm longer fluorescence peaks compared with that of the original dipyrrolo[1,2-*a*:2′,1′-*c*]quinoxaline.

## 1. Introduction

The development of organic fluorophores is an important theme for the advancement of functional materials research. Fused aromatic rings are widely utilized to produce fluorescent materials because of their planar and large π-conjugated structures [1,2,3,4,5,6,7,8,9]. Recently, the fluorophores with longer fluorescence peaks are utilized in the biological probes because the fluorophores with shorter fluorescence peaks are affected by organs. To achieve a longer fluorescent peak, the introduction of substituents is one of the more efficient methods. However, the various electronic properties of substituents lead to various changes in the fluorescent peak, i.e., a bathochromic shift or a small change was obtained by introducing the electron-withdrawing substituent in the case of hypsochromic shift by the electron-donating substituent, and vice versa [10,11,12,13,14]. For example, Hirano et al. reported the substituent effect of 2-phenylimidazo [1,2-*a*]pyrazine-3(7*H*)-ones which showed the bathochromic shift on fluorescence spectra with an increase in the electron-withdrawing property [15]. Furthermore, the donor-acceptor structure is also the fundamental design to change the optical properties by introducing the substituents. It gives longer fluorescence peaks based on a strong intramolecular charge-transfer (ICT) state [16,17]. Such compounds also show the larger Stokes shift. Therefore, those materials have a possibility to be utilized for bioimaging probes [18,19,20]. However, the opposing characteristic of the substituents leads to a different transition. We have reported the fluorescence properties of fused aromatic rings consisting of pyrrole and imidazole [21,22,23,24]. In the investigation of diimidazo [1,2-*a*:2′,1′-*c*]quinoxalines, the *p*-methoxyphenyl substituents at the 3 and 10 positions, we found that they resulted in a large bathochromic shift of a fluorescence peak of 13 nm based on the phenyl substituted compound, whereas a small change in fluorescence peak of 4 nm was obtained from the compound with *p*-trifluoromethylphenyl substituents [22].

The introduction of the electron-donating and electron-withdrawing substituents is also utilized for tuning the electric properties of the substrates. Usually, electron-donating substituents increase the orbital energies, and electron-withdrawing substituents decrease the orbital energies. Thus, by tuning the fluorescence peak with the introduction of substituents, the electric properties were also affected. When we must control both the optical and electric properties, it is a challenging problem to give consideration relating them to each other. For example, the emitting material in the organic light-emitting diode (OLED) is important to tune its luminescent peak as well as HOMO and LUMO energies because hole- and electron-injections are also affected by the HOMO and LUMO energies. Anzenbacher, Jr. et al. examined the OLED properties of substituted tris(8-hydroxyquinoline)aluminum (Alq_3_) complexes [25]. The fluorescence peaks were given a bathochromic shift compared with the original Alq_3_, but the change of HOMO and LUMO energies were varied; the properties of OLED were also affected by the device configurations. Thus, if the materials were developed with the substituent effect on the optical and electric properties separately, it will be useful for molecular design of functional materials that utilize both optical and electric properties.

**Scheme 1:**
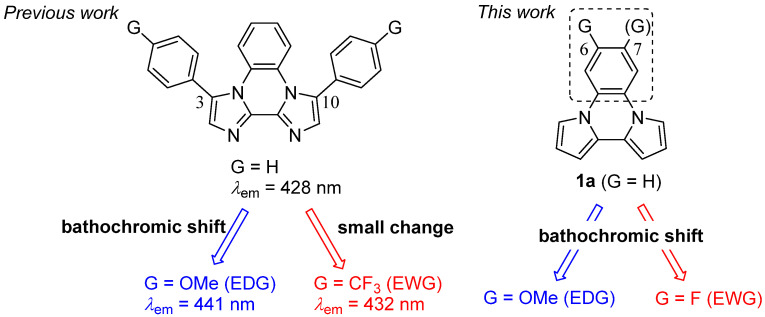
Representation of the difference of fluorescence peaks of diazolo [1,2-*a*:2′,1′-*c*]quinoxalines with substituent(s) on 3,10- or 6,7-positions.

We focused on the substituent effect on the benzene ring in diazolo [1,2-*a*:2′,1′-*c*]quinoxalines, which would be more directly influenced on the optical properties. Herein, we reported the bathochromic shift from the introduction of each electron-donating and electron-withdrawing substituent over 10 nm in dipyrrolo [1,2-*a*:2′,1′-*c*]quinoxalines (**1**) (Scheme 1). Furthermore, based on the (TD)DFT calculation, we found that the change of the orbital energies was typical with increments by the electron-donating substituent and with decrements by the electron-withdrawing substituent.

## 2. Results and Discussions

**Scheme 2:**
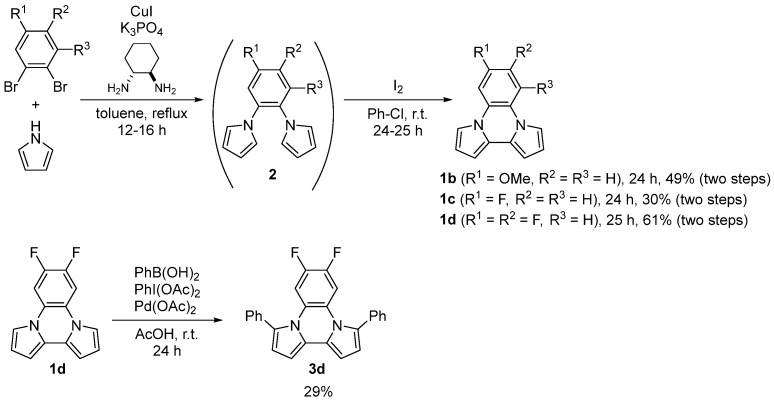
Synthesis of **1** and **3d**.

We chose a dipyrrolo [1,2-*a*:2′,1′-*c*]quinoxaline skeleton to examine the substituent effects because a longer fluorescent wavelength was achieved with dipyrrolo [1,2-*a*:2′,1′-*c*]quinoxaline (**1a**) (*λ*_em_ = 416 nm in THF) than with diimidazo [1,2-*a*:2′,1′-*c*]quinoxaline (*λ*_em_ = 367 nm in THF). The compounds were synthesized by the same procedure mentioned previously [22]. The coupling reaction of pyrrole and substituted dibromobenzene with a copper catalyst produced dipyrrolylbenzenes (**2b**–**d**) by Buchwald amination [26]. After column chromatography, **2b**–**d** was treated with I_2_ to give corresponding **1b**–**d** moderate yield (two steps) (Scheme 2). We also tried to synthesis the other compounds with methyl group at the 6 position or a fluorine substituent at the 5 position. However, we did not succeed in purification through recrystallization and preparative GPC purification. We could not explain the reason why such compounds were difficult to purify. But some contaminations of the positional isomers were supposed from the ^1^H NMR spectra, whose contamination would be derived from the starting dibromobenzene derivatives. In addition, to achieve the longer fluorescence peak, we synthesized **3d** with two phenyl rings at the 3 and 10 positions by the reaction of **1d** with phenyl boric acid in 29% yield [27].

**Figure 1:**
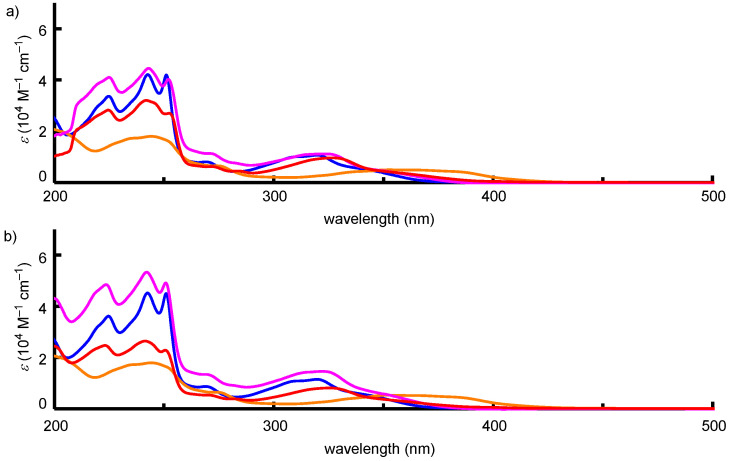
Absorption spectra of **1a** (blue), **1b** (orange), **1c** (purple), and **1d** (red) in (**a**) THF and (**b**) CH_3_CN.

**Figure 2:**
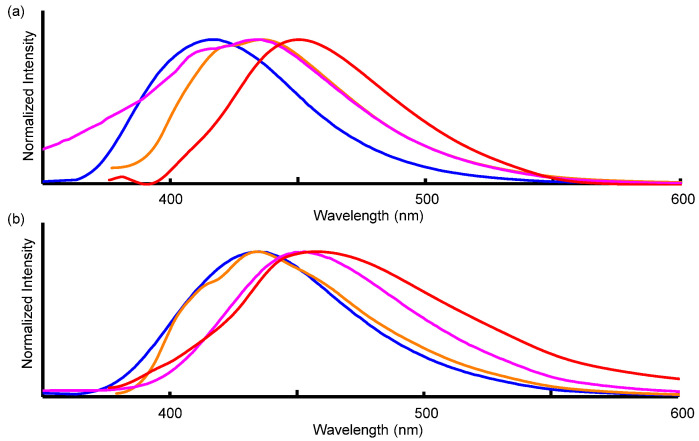
Fluorescence spectra of **1a** (blue), **1b** (orange), **1c** (purple), and **1d** (red) in (**a**) THF and (**b**) CH_3_CN.

Absorption and fluorescence spectra of **1** were measured in THF and CH_3_CN. The results were summarized in Table 1. The absorption peak showed the bathochromic shift in the case of the compound (**1b**) bearing electron-donating group such as methoxy substituents at the 6 position against **1a** (entry 1 vs. entry 2). But a small change of absorption peak was observed in the compounds with fluorine substituent at the 6 position (**1c**) (entry 3). The solvent had little effect on the absorption spectra (Figure 1). Focused on the fluorescence peak, regardless of electron-donating or electron-withdrawing substituents, both compounds showed a bathochromic shift over 10 nm against **1a** in THF (entry 1 vs. entries 2 and 3). By changing the solvent from THF to polar CH_3_CN, more bathochromic shift was obtained from **1c** whereas no solvent effect was found in **1b** (Figure 2). This solvent effect was also observed originally in **1a**. Such a solvent effect would be caused by the charge-transfer transition. Because the pyrrole ring has an electron-rich character, bipyrrole moiety would act as an electron-donating part. By introducing the electron-donating substituent on the benzene ring, charge distribution would be reduced. The Stokes shift also gives the information to their transition. The Stokes shift increases with solvent polarity when the dipole moment is higher in the excited state than in the ground state [28]. In fact, large Stokes shifts (Δ*λ* and Δ*ν*) in CH_3_CN were obtained over 100 nm (over 8000 cm^−1^) in the case of **1a** and **1c.** The value of Stokes shifts of **1a** and **1c** in CH_3_CN were larger than those in THF. Those results also suggested that the fluorescence transition of **1a** and **1c** would be derived from ICT state. However, **1b** provided small change of the Stokes shift by the solvent polarity. Thus, the little effect of charge-transfer transition would be affected on the fluorescence of **1b**. The fluorescence quantum yields (Φ_F_) were also affected by the introduction of the substituent. Especially, **1b** was strongly decreased Φ_F_ compared with **1a** (entry 2 vs. entry 1) although Φ_F_ of **1c** was kept at 0.22 in THF (entry 3). This would be caused by the increment of the vibronic part by introducing substituents such as methoxy groups. We also investigated the compound with two fluorine substituents in the 6 and 7 positions (**1d**). As for the results, a further bathochromic shift of fluorescence peak to reach 458 nm in CH_3_CN was achieved in keeping with the fluorescence quantum yield (entry 4).

**Figure 3:**
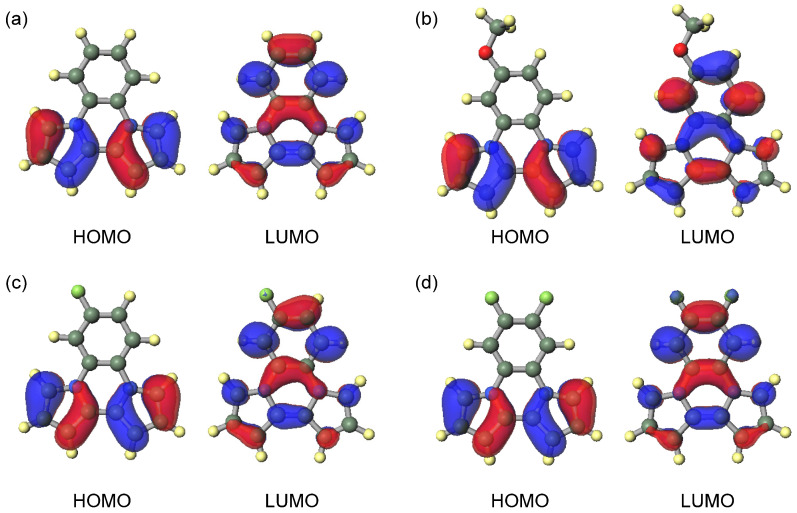
Shape of HOMO and LUMO of (**a**) **1a**, (**b**) **1b**, (**c**) **1c**, and (**d**) **1d** calculated by TDDFT method.

As mentioned in the introduction, it is rational that the compounds (**1c** and **1d**) with electron-withdrawing substituent show the longer fluorescence peak because of their ICT character to account for the bipyrrole moiety as a donor part. However, the reason for the bathochromic shift of the compound bearing the electron-donating substituent (**1b**) is unclear. To clarify the reason for the bathochromic shift on each electronic substituent in fluorescence peak, we examined HOMO and LUMO energies by time-dependent density functional theory (TDDFT) calculation. To discuss the excited state, the optimized structure with minimum energy was estimated by TDDFT calculation. HOMO and LUMO energies, and the differences of each energy, were summarized in Table 2. The molecular orbitals were represented in Figure 3. Every HOMO was localized on bipyrrole moiety, and every LUMO was spread over the molecules. Based on the investigation concerning orbital energy, a good relationship was found between *λ*_em_ in THF and the energy gap between HOMO and LUMO. It is acceptable because the energy gap of HOMO and LUMO is generally attributed to the energy of the transition. In fact, all computed longest transition peaks were attributed from HOMO to LUMO (Table S1). Thus, it is rational to discuss the fluorescence peaks based on HOMO and LUMO. Focused on the energy change in HOMO and LUMO, increased energy against **1a** was obtained in **1b**, which possesses the electron-donating substituent (entry 2). The influence of HOMO energy was also larger than that of LUMO. On the contrary, reduced energy in HOMO and LUMO was obtained in **1c** and **1d** (entries 3 and 4). LUMO was observed to have efficient energy reduction. It is well known to change the orbital energies toward an increase and a decrease by introducing electron-donating and electron-withdrawing substituents, respectively. The small energy gap between HOMO and LUMO, which leads to larger *λ*_em_ in those compounds, would be attributed to the difference of degree of change in HOMO and LUMO energies. The electron-donating substituent increased both HOMO and LUMO energies, but more efficiently increased HOMO energy. In the case of the electron-withdrawing substituent, both energies were reduced, but LUMO energy was more efficiently decreased. As a result, the small energy gap between HOMO and LUMO energies compared with that of **1a** was obtained in all of **1b**, **1c**, and **1d**. From those findings, both electron-donating and electron-withdrawing substituents on dipyrrolo [1,2-a:2′,1′-c]quinoxalines are affected by the bathochromic shift of the fluorescence peak on the optical properties, but the different effect would occur in the case of the electric properties; i.e., the electron-donating substituent increases HOMO and LUMO energies and the electron-withdrawing substituent decreases HOMO and LUMO energies. Such changes of HOMO and LUMO energies were also obtained from the DFT calculation assigned as the ground state (Figure S1). Thus, the change of the electric properties by the introduction of a substituent would be typical both in the ground and excited states.

**Figure 4:**
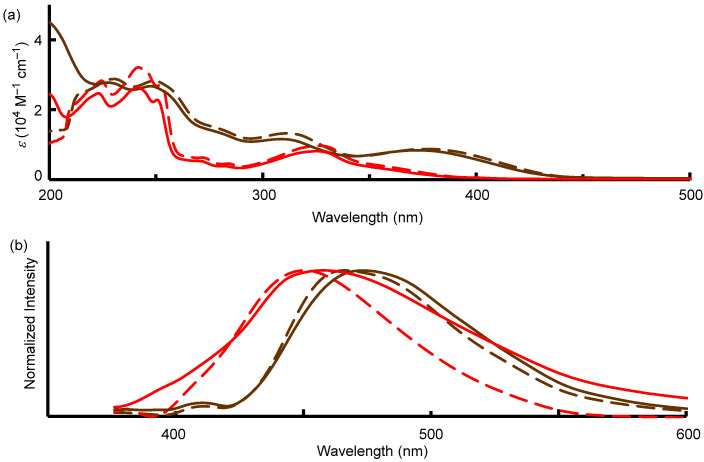
(**a**) Absorption and (**b**) fluorescence spectra of **1d** (red) and **3d** (brown) in THF (dashed line) and CH_3_CN (plane line).

Finally, we attached phenyl rings at the 3 and 10 positions in **1d** to obtain a longer fluorescent peak. This resulted in the observed fluorescence peaks of **3d** at 466 nm and 473 nm in THF and CH_3_CN, respectively (Table 1, entry 5) (Figure 4). Those values were 40–50 nm larger than the original **1a**. The influence of the introduction of phenyl rings at 3 and 10 positions on the fluorescence peak was estimated as bathochromic shift about 20 nm compared with **1d** in each solvent. In the case of the original **1a**, the compound bearing two phenyl rings at 3 and 10 positions (3,10-diphenyldipyrrolo [1,2-*a*:2′,1′-*c*]quinoxaline) showed a longer fluorescence peak of approximately 30 nm (*λ*_em_ = 445 nm in THF) [21]. Thus, the effect of additional phenyl rings at the 3 and 10 positions was reduce by the introduction of the fluorine substituents on benzene ring in dipyrrolo [1,2-*a*:2′,1′-*c*]quinoxaline. Interestingly, the fluorescence quantum yield was increased compared with **1d**. Such effects were also observed in diimidazo [1,2-*a*:2′,1′-*c*]quinoxalines [22]. Thus, it would stand to reason that the steric restriction of vibronic motion would occur by introducing phenyl ring.

## 3. Materials and Methods

### 3.1. General Information

Melting points were determined with Yanaco MP-J3 and values were uncorrected. NMR spectra were recorded at 400 MHz (proton) (100 MHz (carbon-13)) on Bruker AVANCE III-400M. Chemical shifts (*δ*) of ^1^H NMR were expressed in parts per million downfield or upfield from tetramethylsilane in CDCl_3_ as an internal standard. Multiplicities are indicated as s (singlet), d (doublet), t (triplet), m (multiplet), and coupling constants (*J*) are reported in hertz units. Chemical shifts (*δ*) of ^13^C{^1^H} NMR are expressed in parts per million downfield or upfield from CDCl_3_ (*δ* = 77.0) as an internal standard. Infrared (IR) spectra were recorded on a JASCO FT/IR-460 plus spectrometer. Mass spectra were carried out on THERMO Fisher Exactive in the Center for Analytical Instrumentation of Chiba University. Anhydrous toluene was distilled from CaH_2_ and was stored with MS 4 Å. All other commercially available materials were used without further purification. The reactions were performed under nitrogen or argon atmosphere unless otherwise noted.

#### 3.1.1. Preparation of 1,2-Dibromo-4-methoxybenzene [29]

*N*-bromosuccinimide (0.889 g, 4.99 mmol) and 1 *M* HCl (50 μL) was added to a solution of 3-bromoanisole (0.63 mL, 5.0 mmol) in acetone (10 mL). The mixture was stirred for 20 min at room temperature. After the disappearance of yellow color, the reaction mixture was evaporated in vacuo. The residue was extracted with CHCl_3_ (20 mL × 3). The organic layer was dried with MgSO_4_. After filtration and evaporation, 1,2-dibromo-4-methoxybenzene (1.171 g, 4.40 mmol) was obtained in 88% yield as colorless oil. ^1^H NMR (CDCl_3_, 400 MHz): *δ* 3.77 (s, 3H), 6.72 (dd, *J* = 2.9 and 8.9 Hz, 1H), 7.16 (d, *J* = 2.9 Hz, 1H), 7.46 (d, *J* = 8.9 Hz, 1H).

#### 3.1.2. Synthesis of Substituted Dipyrrolo [1,2-*a*:2′,1′-*c*]quinoxaline (**1b**–**d**)

6-Methoxydipyrrolo [1,2-*a*:2′,1′-*c*]quinoxaline (**1b**): A mixture of 1,2-dibromo-4-methoxybenzene (1.46 g, 5.49 mmol), pyrrole (1.0 mL, 14.4 mmol), CuI (95.0 mg, 0.499 mmol), K_3_PO_4_ (4.22 g, 19.9 mmol), and *trans*-1,2-cyclohexanediamine (0.60 mL, 5.0 mmol) in toluene (10 mL) was stirred for 12 h under refluxing conditions. After being cooled to room temperature, the reaction mixture was filtered with a plug of Celite washing with EtOAc. After evaporation in vacuo, the residual mixture was subjected to column chromatography on silica-gel (*n*-hexane:EtOAc = 6:1) to give the product (1.507 g). The product was dissolved in chlorobenzene (4 mL). To the solution was added a solution of iodine (0.834 g, 3.29 mmol) in chlorobenzene (6 mL) in a period of 5 min. After being stirred for 24 h at room temperature, to the reaction mixture was added saturated aqueous Na_2_S_2_O_3_ solution (10 mL) and acetone (10 mL) to dissolve the precipitate. The combined mixture was extracted with CHCl_3_ (20 mL × 3). The organic layer was dried with MgSO_4_. After filtration and evaporation, the residue was subjected to column chromatography on SiO_2_ (*n*-hexane:EtOAc = 8:1) to give 6-methoxydipyrrolo [1,2-*a*:2′,1′-*c*]quinoxaline (0.638 g, 2.70 mmol, 49% (two steps)) as pale-yellow solid: m.p. = 91–92 °C (*n*-hexane/CHCl_3_). ^1^H NMR (CDCl_3_, 400 MHz): *δ* 3.91 (s, 3H), 6.52–6.58 (m, 4H), 6.85 (dd, *J* = 2.7 and 9.0 Hz, 1H), 7.21 (d, *J* = 2.6 Hz, 1H), 7.42 (dd, *J* = 1.5 and 2.6 Hz, 2H), 7.64 (d, *J* = 8.9 Hz, 1H). ^13^C{^1^H} NMR (CDCl_3_, 100 MHz): *δ* 55.7, 100.6, 100.7, 101.4, 110.2, 111.6, 111.7, 111.9, 112.4, 116.4. 119.8, 123.5, 124.4, 126.4, 156.6. IR (KBr): *ν* 3854, 3802, 3745, 3676, 1700, 1654, 1522, 768, 684 cm^−1^. HRMS (ESI) *m/z*: [M−H]¯ calcd for C_15_H_11_N_2_O 235.0877; found 235.0884. UV-Vis absorption: *λ*_abs_ [ε (M^−1^ cm^−1^)] 367 [4800], 279(sh) [5307], 244.5 [18,020] nm (3.0 × 10^−5^ M in THF); 369 [4900], 278.5(sh) [5393], 244.5 [17,895] nm (3.0 × 10^−5^ M in CH_3_CN).

6-Fluorodipyrrolo [1,2-*a*:2′,1′-*c*]quinoxaline (**1c**): Yield 30% (two steps) (66.5 mg) as white solid: m.p. = 107–109 °C (*n*-hexane/CHCl_3_). ^1^H NMR (CDCl_3_, 400 MHz): *δ* 6.52–6.54 (m, 2H), 6.57 (t, *J* = 3.0 Hz, 1H), 6.58 (t, *J* = 3.6 Hz, 1H), 6.99 (ddd, *J* = 2.7, 7.8 Hz and *J*_H-C-C-F_ = 9.0 Hz, 1H), 7.36 (dd, *J* = 1.4 and 3.0 Hz, 1H), 7.40 (dd, *J* = 2.7 Hz and *J*_H-C-C-F_ = 9.5 Hz, 1H), 7.42 (m, 1H), 7.66 (dd, *J*_H-C-C-C-F_ = 5.1 Hz and *J* = 9.1 Hz, 1H). ^13^C{^1^H} NMR (CDCl_3_, 100 MHz): *δ* 101.3 (d, *J*_C-C-F_ = 27.1 Hz), 102.6 (d, *J*_C-C-F_ = 27.7 Hz), 111.1, 111.4, 111.9, 112.1, 112.3, 112.8, 116.5 (d, *J*_C-C-C-F_ = 9.2 Hz), 122.1 (d, *J*_C-C-C-C-F_ = 2.2 Hz), 124.2, 123.6, 126.4 (d, *J*_C-C-C-F_ = 10.3 Hz), 159.3 (d, *J*_C-F_ = 243.5 Hz). IR (KBr): *ν* 3113, 2359, 1353, 1285, 1626, 1588, 1520, 1191, 768, 682 cm^−1^. HRMS (ESI) *m/z*: [M+H]^+^ calcd for C_14_H_10_FN_2_ 225.0823; found 225.0826. UV-Vis absorption: *λ*_abs_ [ε (M^−1^ cm^−1^)] 322 [11,200], 275.5(sh) [8638], 252 [40,240], 243 [44,583] nm (3.0 × 10^−5^ M in THF); 322 [15,600], 269(sh) [13,341], 251 [011,49], 242 [53,256] nm (3.0 × 10^−5^ M in CH_3_CN).

6,7-Difluorodipyrrolo [1,2-*a*:2′,1′-*c*]quinoxaline (**1d**): Yield 61% (two steps) (0.566 g) as pale-green solid: m.p. = 116–117 °C (*n*-hexane/CHCl_3_). ^1^H NMR (CDCl_3_, 400 MHz): *δ* 6.54 (dd, *J* = 1.4 and 3.7 Hz, 2H), 6.59 (dd, *J* = 3.0 and 3.6 Hz, 2H), 7.32 (dd, *J* = 1.5 and 3.0 Hz, 2H), 7.51 (t, *J*_H-C-C-F_ = 8.5 Hz, 2H). ^13^C{^1^H} NMR (CDCl_3_, 100 MHz): *δ* 101.6, 104.5 (dd, *J*_C-C-C-F_ = 9.0 Hz and *J*_C-C-F_ = 14.5 Hz), 112.1, 112.9, 121.78 (t, *J*_C-C-C-F_ = 5.4 Hz), 123.8, 146.9 (dd, *J*_C-C-F_ = 15.6 Hz and *J*_C-F_ = 248.0 Hz). IR (KBr): *ν* 3744, 3675, 3412, 1685, 1598, 1340, 1265, 1227, 1083, 838 cm^−1^. HRMS (ESI) *m/z*: [M+H]^+^ calcd for C_14_H_9_F_2_N_2_ 243.0728; found 243.0729. UV-Vis absorption: *λ*_abs_ [ε (M^−1^ cm^−1^)] 325 [9500], 283.5 [4546], 272 [6252], 252 [27,013], 242 [32,073] nm (3.0 × 10^−5^ M in THF); 324 [8100], 282 [3810], 269.5(sh) [5297], 250.5 [22,736], 241.5 [26,341] nm (3.0 × 10^−5^ M in CH_3_CN).

#### 3.1.3. Synthesis of 6,7-Difluoro-3,10-diphenyldipyrrolo [1,2-*a*:2′,1′-*c*]quinoxaline (**3d**)

PhI(OAc)_2_ (0.602 g, 1.87 mmol) and PhB(OH)_2_ (0.230 g, 1.89 mmol) was added to acetic acid (9 mL). After being stirred for 20 min at room temperature, 1d (0.139 g, 0.574 mmol) was added to the mixture. After being stirred for 15 min, Pd(OAc)_2_ (20 mg, 0.089 mmol) was added and then the whole was stirred for 24 h at room temperature. The reaction mixture was filtered through a plug of Celite. After evaporation in vacuo, the residue was added to water (10 mL), and was extracted with CHCl_3_ (10 mL × 3). The organic layer was dried with MgSO_4_. After filtration and evaporation, the residue was subjected to column chromatography on SiO_2_ (*n*-hexane:EtOAc = 10:1) to give 6,7-difluoro-3,10-diphenyldipyrrolo [1,2-*a*:2′,1′-*c*]quinoxaline (65.3 mg, 0.166 mmol, 29%) as yellow solid: m.p. = 167–168 °C (*n*-hexane/CHCl_3_). ^1^H NMR (CDCl_3_, 400 MHz): *δ* 6.56 (d, *J* = 3.7 Hz, 2H), 6.63 (d, *J* = 3.8 Hz, 2H), 7.10 (t, *J*_H-C-C-F_ = 10.1 Hz, 2H), 7.39 (tt, *J* = 1.3 and 7.2 Hz, 2H), 7.46 (diffused t, *J* = 7.5 Hz, 4H), 7.51 (diffused d, *J* = 6.9 Hz, 4H). ^13^C{^1^H} NMR (CDCl_3_, 100 MHz): *δ* 102.0, 108.5 (dd, *J*_C-C-C-F_ = 9.5 Hz and *J*_C-C-F_ = 15.4 Hz), 116.2, 124.0 (t, *J*_C-C-C-F_ = 5.9 Hz), 127.4, 127.8, 128.2, 128.3, 129.0, 131.0, 133.4, 145.5 (dd, *J*_C-C-F_ = 15.4 Hz and *J*_C-F_ = 247.2 Hz). IR (KBr): *ν* 3854, 3821, 3676, 3629, 3421, 1700, 1685, 1654, 1598, 1517 cm^−1^. HRMS (ESI) *m/z*: [M+H]^+^ calcd for C_26_H_17_F_2_N_2_ 395.1354; found 395.1343. UV-Vis absorption: *λ*_abs_ [ε (M^−1^ cm^−1^)] 377 [10,600], 312 [13,210], 249 [28,198] nm (3.0 × 10^−5^ M in THF); 375 [8700], 309 [11,565], 247.5 [26,700] nm (3.0 × 10^−5^ M in CH_3_CN).

### 3.2. Measurement of Absorption and Fluorescence Spectra

The materials measuring the optical properties were purified by recrystallization from CHCl_3_ and *n*-hexane. UV-Vis spectra were measured with quartz cell (1 cm × 1 cm) on a JASCO V-570 spectrophotometer. Fluorescence spectra were measured with quartz cell (1 cm × 1 cm) on a JASCO FP-6600 spectrofluorometer.

### 3.3. DFT Calculation Method

Shape of orbitals, and HOMO and LUMO energies were calculated by TDDFT/ωB97XD/6-31+G(d,p) level of theory with the Gaussian 16W program version 1.1 [30]. The optimized structure was also obtained from TDDFT calculation by ωB97XD/6-31+G(d,p) level of theory.

## 4. Conclusions

In conclusion, the introduction of a substituent on benzene ring in dipyrrolo [1,2-*a*:2′,1′-*c*]quinoxaline was efficient to give longer fluorescence peak. Both substituents with electron-donating and electron-withdrawing character were available to make bathochromic shift. Especially, similar change was observed in THF from each compound with electron-donating methoxy group or electron-withdrawing fluorine substituent. Such substituent effect would be unique in a dipyrrolo [1,2-*a*:2′,1′-*c*]quinoxalines skeleton. In the case of the electron-withdrawing substituent, the solvent effect was also observed, which implied that the fluorescence caused by ICT state.

The reason for the shift of fluorescence peak was explainable by change of the orbital energies. Electron-donating substituents increased both HOMO and LUMO energies, especially HOMO energy. In a complementary style, electron-withdrawing substituents decreased both HOMO and LUMO energies, especially LUMO energy. As a result, a smaller energy gap compared with the original substrate was achieved in each electron-donating and electron-withdrawing substituent. It means that the different effect against optical and electric properties was obtained by using dipyrrolo [1,2-*a*:2′,1′-*c*]quinoxaline structure.

An additional introduction of substituent such as fluorine at 7 position or phenyl ring at 3 and 10 position on dipyrrolo [1,2-*a*:2′,1′-*c*]quinoxalines is efficient to give the longer fluorescence peaks. As a result, we obtained the fluorescence peak at 473 nm from **3d**, whose value is 39 nm larger than that of non-substituted compounds (**1a**). These findings will serve as guidelines in the design of novel fluorophores with longer fluorescence.

## Figures and Tables

**Table 1:** Optical Properties of **1** and **3f** in THF and CH_3_CN.

Entry	Compound	*λ*_abs_ (nm) [ε (M^−1^ cm^−1^)] ^1^		*λ*_em_ (nm) ^2^ [Φ_F_] ^3^		Δ*λ* (nm) ^4^ [Δ*ν* (cm^−1^) ^5^]	
		In THF	In CH_3_CN	In THF	In CH_3_CN	In THF	In CH_3_CN
1	**1a**	321 [10,600]	320 [10,700]	416 [0.43]	434 [0.17]	95 [7114]	114 [8209]
2	**1b**	367 [4800]	369 [4900]	435 [0.01]	434 [0.02]	68 [4259]	65 [4059]
3	**1c**	322 [11,200]	322 [15,600]	434 [0.22]	453 [0.12]	112 [8014]	131 [8981]
4	**1d**	325 [9500]	324 [8100]	449 [0.25]	458 [0.21]	124 [8498]	134 [9030]
5	**3d**	377 [10,600]	375 [8700]	466 [0.35]	473 [0.32]	89 [5066]	98 [5525]

^1^ Concentration: 3.0 × 10^−5^ M. ^2^ Concentration: 3.0 × 10^−7^ M. Excited at λ_abs_. ^3^ Determined using *p*-terphenyl (Φ_F_ = 0.87, 265 nm) as a standard. ^4^ Δ*λ* = *λ*_em_ − *λ*_abs_. ^5^ Δ*ν* = 1/*λ*_abs_ − 1/*λ*_em_.

**Table 2:** HOMO and LUMO energies and the differences of each energy of **1** ^1^.

Entry	Compound	HOMO Energy (eV)	Difference of HOMO Energy against 1a (eV)	LUMO Energy (eV)	Difference of LUMO Energy against 1a (eV)	Energy Gap between HOMO and LUMO (eV)	*λ*_em_ (nm) in THF
1	**1a**	−6.5830	-	6.8151	-	6.8151	416
2	**1b**	−6.5122	0.0708	0.2653	0.0332	6.7775	435
4	**1c**	−6.7092	−0.1262	−0.0063	−0.2384	6.7029	434
6	**1d**	−6.8233	−0.2403	−0.1973	−0.4294	6.6260	449

^1^ Calculated by TDDFT/ωB97XD/6-31+G(d,p) with the optimized structure.

## Data Availability

Not applicable.

## Supplementary Materials

The following supporting information can be downloaded at: https://www.mdpi.com/article/10.3390/molecules28072896/s1, Computed three longest transition peaks of **1a**–**d** in the excited singlet state (Table S1), Shape and energies of HOMO and LUMO of **1a**–**d** calculated by DFT method (Figure S1), Energies, and Cartesian coordinates of **1a**–**d** by TDDFT calculation, and copies of ^1^H and ^13^C NMR spectra and HRMS charts for new compounds (**1b**–**d** and **3d**).

## Sample Availability

Sample of the compounds are not available from the authors.
