# Supplementary material for: Bathochromic Shift of Fluorescence Peak in Dipyrrolo[1,2-a:2′,1′-c]quinoxaline by Introducing Each of Electron-Donating and Electron-Withdrawing Substituent
Source: Molecules. 2023 Mar 23;28(7):2896. doi: 10.3390/molecules28072896 (PMC10096015; doi:10.3390/molecules28072896)

# Electronic Supporting Information for the article entitled

# Bathochromic Shift of Fluorescence Peak in Dipyrrolo[1,2-*a*:2′,1′-*c*]quinoxaline by Introducing Each of Electron-Donating and Electron-Withdrawing Substituent

Shoji Matsumoto,* Makoto Takamori, and Motohiro Akazome

Department of Applied Chemistry and Biotechnology, Graduate School of Engineering, Chiba University, 1-33 Yayoi-cho, Inage-ku, Chiba 263-8522, Japan

E-mail address: smatsumo@faculty.chiba-u.jp

Table of Contents

1. Computed transition peaks of **1a-d** in the excited state (TDDFT calculation) ……..S2
2. HOMO and LUMO energies of **1a-d** (DFT calculation) ……..S3
3. Energies, and Cartesian coordinates of **1a-d** optimized by DFT and TDDFT calculation ……..S4
4. $^{1}$H and $^{13}$C NMR spectra, and HRMS charts for new compounds (**1b-d** and **3d**) ……..S8

**Table S1.** Computed three longest transition peaks of **1a-d** in the excited singlet state calculated by TDDFT (nstate = 10) using optimized structure (TDDFT (nstate = 1) for the excited state) at TDDFT/ωB97XD/6-31+G(d,p) level

| Compound | Transition | Band gap (eV) | Wavelength (nm) | Oscillator strength |
|---|---|---|---|---|
| **1a** | HOMO → LUMO (0.69563) | 3.4559 | 358.76 | 0.1507 |
| | HOMO-1 → LUMO (0.20414)<br>HOMO → LUMO+1 (0.66320) | 4.1183 | 301.06 | 0.0015 |
| | HOMO-2 → LUMO+1 (-0.15826)<br>HOMO-1 → LUMO (0.62692)<br>HOMO → LUMO+1 (-0.21684) | 4.5444 | 272.83 | 0.2249 |
| **1b** | HOMO → LUMO (0.69702) | 3.4219 | 362.32 | 0.1426 |
| | HOMO-1 → LUMO (0.50409)<br>HOMO → LUMO+1 (-0.45588) | 4.2142 | 294.21 | 0.0478 |
| | HOMO-2 → LUMO+1 (-0.11140)<br>HOMO-1 → LUMO (0.43801)<br>HOMO → LUMO+1 (0.51652) | 4.4677 | 277.51 | 0.2229 |
| **1c** | HOMO → LUMO (0.69829) | 3.3497 | 370.13 | 0.1234 |
| | HOMO-1 → LUMO (0.27068)<br>HOMO → LUMO+1 (0.63558) | 4.1404 | 299.45 | 0.0089 |
| | HOMO-2 → LUMO+1 (0.13571)<br>HOMO-1 → LUMO (0.60629)<br>HOMO → LUMO+1 (-0.28423) | 4.4839 | 276.51 | 0.2385 |
| **1d** | HOMO 62 → LUMO 63 (0.69979) | 3.2895 | 376.91 | 0.1122 |
| | HOMO-1 → LUMO (-0.30893)<br>HOMO → LUMO+1 (0.62104) | 4.1387 | 299.57 | 0.0029 |
| | HOMO-2 → LUMO+1 (-0.12028)<br>HOMO-1 → LUMO (0.59214)<br>HOMO → LUMO+1 (0.32296) | 4.4321 | 279.74 | 0.2516 |

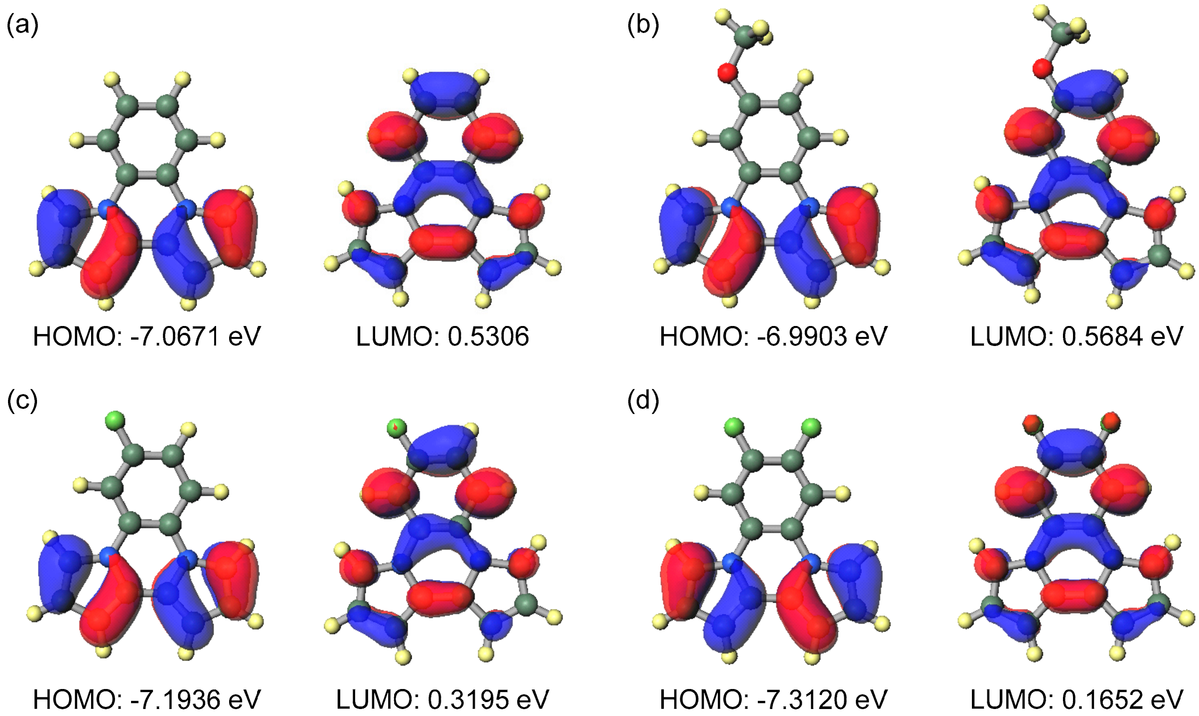


**Figure S1.** Shape and energies of HOMO and LUMO of (a) **1a**, (b) **1b**, (c) **1c**, and (d) **1d** calculated by DFT/ωB97XD/6-31+G(d,p) level of theory. Optimized structure was estimated by DFT/ωB97XD/6-31+G(d,p) level of theory.

**Dipyrrolo[1,2-*a*:2′,1′-*c*]quinoxaline (1a) optimized by TDDFT calculation**

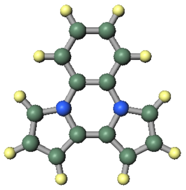

Total energy: -648.812490362 A.U.

Cartesian coordinates:

| | | | | | | | |
|---|---|---|---|---|---|---|---|
| C1 | 1.490552 | -0.689215 | -0.000005 | C12 | -2.185345 | -1.415488 | 0.000027 |
| N1 | 0.261564 | -1.356826 | 0.000008 | H1 | -0.299057 | 3.416155 | -0.000018 |
| C2 | -0.978993 | -0.700576 | 0.000011 | H2 | 2.368202 | 3.876852 | -0.000034 |
| C3 | -0.978975 | 0.700582 | 0.000001 | H3 | 2.368183 | -3.876862 | 0.000005 |
| N2 | 0.261564 | 1.356824 | -0.000010 | H4 | -0.299073 | -3.416151 | 0.000024 |
| C4 | 1.490561 | 0.689208 | -0.000013 | C13 | -3.401351 | -0.687831 | 0.000028 |
| C5 | 0.500779 | 2.694910 | -0.000019 | H5 | -2.197506 | -2.496838 | 0.000036 |
| C6 | 1.894973 | 2.905813 | -0.000027 | H6 | -2.197507 | 2.496849 | -0.000010 |
| C7 | 2.522044 | 1.675816 | -0.000025 | C14 | -3.401338 | 0.687843 | 0.000014 |
| C8 | 2.522031 | -1.675824 | -0.000008 | H7 | -4.339899 | -1.232372 | 0.000040 |
| C9 | 1.894957 | -2.905822 | 0.000004 | H8 | -4.339885 | 1.232389 | 0.000015 |
| C10 | 0.500770 | -2.694914 | 0.000014 | H9 | 3.581376 | 1.467424 | -0.000030 |
| C11 | -2.185348 | 1.415499 | 0.000000 | H10 | 3.581363 | -1.467432 | -0.000017 |

**6-Methoxydipyrrolo[1,2-*a*:2′,1′-*c*]quinoxaline (1b) optimized by TDDFT calculation**

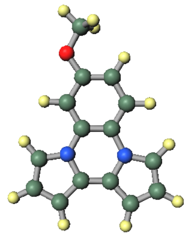

Total energy: -763.305540663 A.U.

Cartesian coordinates:

| | | | | | | | |
|---|---|---|---|---|---|---|---|
| C1 | 2.278960 | -0.462446 | -0.000026 | H2 | 2.388823 | 4.186398 | -0.000117 |
| N1 | 1.186228 | -1.327532 | 0.000006 | H3 | 3.684304 | -3.455790 | 0.000009 |
| C2 | -0.155160 | -0.886655 | 0.000020 | H4 | 0.975929 | -3.448476 | 0.000044 |
| C3 | -0.380577 | 0.498634 | -0.000008 | C13 | -2.556442 | -1.271114 | 0.000068 |
| N2 | 0.726140 | 1.351185 | -0.000041 | H5 | -1.062580 | -2.845918 | 0.000079 |
| C4 | 2.050825 | 0.898606 | -0.000054 | H6 | -1.907532 | 2.066089 | -0.000037 |
| C5 | 0.741489 | 2.709818 | -0.000063 | C14 | -2.768141 | 0.092819 | 0.000037 |
| C6 | 2.084111 | 3.150071 | -0.000093 | H7 | -3.377866 | -1.975212 | 0.000093 |
| C7 | 2.905581 | 2.042335 | -0.000088 | O1 | -3.995820 | 0.684091 | 0.000039 |
| C8 | 3.461828 | -1.259084 | -0.000034 | H8 | 3.984782 | 2.011777 | -0.000108 |
| C9 | 3.053677 | -2.578920 | 0.000001 | H9 | 4.470136 | -0.873185 | -0.000057 |
| C10 | 1.644639 | -2.603908 | 0.000021 | C15 | -5.130924 | -0.150827 | 0.000111 |
| C11 | -1.689875 | 1.007740 | -0.000001 | H10 | -5.160205 | -0.785627 | -0.894715 |
| C12 | -1.222063 | -1.776023 | 0.000059 | H11 | -5.160125 | -0.785581 | 0.894970 |
| H1 | -0.168029 | 3.287114 | -0.000053 | H12 | -5.995227 | 0.513815 | 0.000132 |

**6-Fluorodipyrrolo[1,2-*a*:2′,1′-*c*]quinoxaline (1c) optimized by TDDFT calculation**

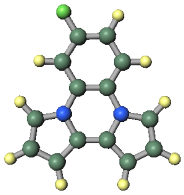

Total energy: -748.024120848 A.U.

Cartesian coordinates:

| | | | | | | | |
|---|---|---|---|---|---|---|---|
| C1 | 1.920221 | -0.408349 | -0.000001 | C12 | -1.527719 | -1.854140 | 0.000001 |
| N1 | 0.859505 | -1.309649 | 0.000000 | H1 | -0.653885 | 3.252424 | 0.000001 |
| C2 | -0.498585 | -0.916542 | 0.000000 | H2 | 1.869986 | 4.241547 | -0.000002 |
| C3 | -0.766544 | 0.458402 | 0.000001 | H3 | 3.428159 | -3.350688 | 0.000000 |
| N2 | 0.307896 | 1.350248 | 0.000000 | H4 | 0.721286 | -3.436048 | 0.000000 |
| C4 | 1.646351 | 0.944953 | -0.000002 | C13 | -2.878697 | -1.394312 | 0.000001 |
| C5 | 0.275096 | 2.707116 | 0.000000 | H5 | -1.325745 | -2.916020 | 0.000001 |
| C6 | 1.601945 | 3.195235 | -0.000002 | H6 | -2.360323 | 1.976468 | 0.000002 |
| C7 | 2.460727 | 2.117197 | -0.000001 | C14 | -3.109464 | -0.045723 | 0.000001 |
| C8 | 3.129152 | -1.163192 | -0.000001 | H7 | -3.707893 | -2.090044 | 0.000001 |
| C9 | 2.767480 | -2.496349 | 0.000000 | F1 | -4.387810 | 0.403786 | 0.000001 |
| C10 | 1.360635 | -2.569238 | -0.000001 | H8 | 3.540334 | 2.124534 | -0.000002 |
| C11 | -2.100107 | 0.928680 | 0.000002 | H9 | 4.123616 | -0.742860 | -0.000002 |

**6,7-Difluorodipyrrolo[1,2-*a*:2′,1′-*c*]quinoxaline (1d) optimized by TDDFT calculation**

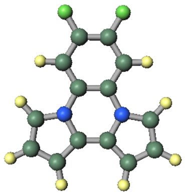

Total energy: -847.228319917 A.U.

Cartesian coordinates:

| | | | | | | | |
|---|---|---|---|---|---|---|---|
| C1 | 2.076449 | 0.690334 | 0.000000 | C12 | -1.590351 | 1.425033 | 0.000002 |
| N1 | 0.851927 | 1.357547 | 0.000000 | H1 | 0.288498 | -3.412724 | 0.000000 |
| C2 | -0.389307 | 0.698497 | 0.000001 | H2 | 2.957724 | -3.875244 | -0.000002 |
| C3 | -0.389315 | -0.698496 | 0.000001 | H3 | 2.957715 | 3.875248 | 0.000000 |
| N2 | 0.851930 | -1.357549 | 0.000001 | H4 | 0.288489 | 3.412718 | 0.000000 |
| C4 | 2.076447 | -0.690334 | 0.000000 | C13 | -2.795470 | 0.682257 | 0.000000 |
| C5 | 1.088941 | -2.692026 | 0.000000 | H5 | -1.637676 | 2.503218 | 0.000002 |
| C6 | 2.484344 | -2.904520 | -0.000001 | H6 | -1.637671 | -2.503217 | -0.000001 |
| C7 | 3.108333 | -1.674353 | 0.000000 | C14 | -2.795474 | -0.682257 | -0.000001 |
| C8 | 3.108333 | 1.674357 | 0.000000 | F1 | -3.959660 | 1.362413 | 0.000000 |
| C9 | 2.484342 | 2.904520 | 0.000000 | F2 | -3.959663 | -1.362415 | -0.000003 |
| C10 | 1.088937 | 2.692025 | 0.000000 | H7 | 4.167329 | -1.464301 | 0.000000 |
| C11 | -1.590348 | -1.425033 | 0.000000 | H8 | 4.167329 | 1.464302 | 0.000000 |

**Dipyrrolo[1,2-*a*:2′,1′-*c*]quinoxaline (1a) optimized by DFT calculation**

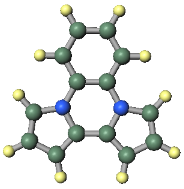

Total energy: -648.825408476 A.U.

Cartesian coordinates:

| | | | | | | | |
|---|---|---|---|---|---|---|---|
| C1 | 1.487997 | 0.718509 | 0.000006 | C12 | -2.175009 | 1.389537 | -0.000029 |
| N1 | 0.270025 | 1.380690 | -0.000013 | H1 | -0.300890 | -3.457162 | 0.000019 |
| C2 | -0.958883 | 0.703721 | -0.000015 | H2 | 2.354032 | -3.898327 | 0.000028 |
| C3 | -0.959146 | -0.703394 | 0.000000 | H3 | 2.356337 | 3.897185 | -0.000004 |
| N2 | 0.269328 | -1.380678 | 0.000013 | H4 | -0.298503 | 3.457629 | -0.000029 |
| C4 | 1.487760 | -0.719189 | 0.000015 | C13 | -3.377565 | 0.697663 | -0.000028 |
| C5 | 0.503858 | -2.740873 | 0.000020 | H5 | -2.181753 | 2.472721 | -0.000042 |
| C6 | 1.861965 | -2.936509 | 0.000023 | H6 | -2.183789 | -2.471825 | 0.000016 |
| C7 | 2.489923 | -1.662809 | 0.000022 | C14 | -3.377803 | -0.695803 | -0.000012 |
| C8 | 2.490879 | 1.661620 | 0.000014 | H7 | -4.312115 | 1.247826 | -0.000040 |
| C9 | 1.863791 | 2.935587 | -0.000003 | H8 | -4.312839 | -1.245397 | -0.000009 |
| C10 | 0.505423 | 2.740425 | -0.000015 | H9 | 3.549705 | -1.455864 | 0.000026 |
| C11 | -2.175888 | -1.388625 | 0.000002 | H10 | 3.550528 | 1.453959 | 0.000028 |

**6-Methoxydipyrrolo[1,2-*a*:2′,1′-*c*]quinoxaline (1b) optimized by DFT calculation**

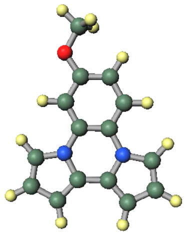

Total energy: -763.318521311 A.U.

Cartesian coordinates:

| | | | | | | | |
|---|---|---|---|---|---|---|---|
| C1 | -2.285285 | -0.480698 | 0.000079 | H2 | -2.344553 | 4.217374 | -0.000254 |
| N1 | -1.201405 | -1.341853 | 0.000005 | H3 | -3.689099 | -3.461865 | 0.000295 |
| C2 | 0.127669 | -0.884087 | -0.000095 | H4 | -0.996234 | -3.483724 | 0.000163 |
| C3 | 0.371619 | 0.503786 | 0.000024 | C13 | 2.521030 | -1.285708 | -0.000238 |
| N2 | -0.722629 | 1.379824 | -0.000002 | H5 | 1.043358 | -2.824625 | -0.000625 |
| C4 | -2.036908 | 0.935954 | 0.000023 | H6 | 1.893952 | 2.037076 | 0.000372 |
| C5 | -0.720265 | 2.760228 | -0.000033 | C14 | 2.754260 | 0.090749 | 0.000168 |
| C6 | -2.024383 | 3.185493 | -0.000162 | H7 | 3.334595 | -1.999817 | -0.000773 |
| C7 | -2.861576 | 2.037697 | -0.000143 | O1 | 3.984626 | 0.665167 | 0.000453 |
| C8 | -3.437069 | -1.237228 | 0.000081 | H8 | -3.941169 | 2.015497 | -0.000212 |
| C9 | -3.038888 | -2.599041 | 0.000182 | H9 | -4.444932 | -0.849607 | 0.000096 |
| C10 | -1.665761 | -2.639406 | 0.000095 | C15 | 5.116640 | -0.179881 | -0.000144 |
| C11 | 1.678899 | 0.976333 | 0.000186 | H10 | 5.143460 | -0.812581 | 0.895525 |
| C12 | 1.211617 | -1.754482 | -0.000352 | H11 | 5.143429 | -0.811350 | -0.896625 |
| H1 | 0.195845 | 3.327268 | -0.000082 | H12 | 5.982982 | 0.480975 | 0.000451 |

**6-Fluorodipyrrolo[1,2-*a*:2′,1′-*c*]quinoxaline (1c) optimized by DFT calculation**

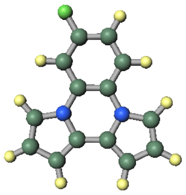

Total energy: -748.03783448 A.U.

Cartesian coordinates:

| | | | | | | | |
|---|---|---|---|---|---|---|---|
| C1 | -1.932344 | -0.413359 | -0.000001 | C12 | 1.506415 | -1.843434 | -0.000004 |
| N1 | -0.883246 | -1.319280 | -0.000004 | H1 | 0.706061 | 3.287511 | -0.000009 |
| C2 | 0.461843 | -0.918082 | -0.000002 | H2 | -1.793582 | 4.282864 | 0.000003 |
| C3 | 0.759665 | 0.458009 | 0.000001 | H3 | -3.456404 | -3.334760 | 0.000005 |
| N2 | -0.293670 | 1.379113 | 0.000001 | H4 | -0.769296 | -3.469489 | -0.000005 |
| C4 | -1.626209 | 0.991323 | 0.000000 | C13 | 2.831449 | -1.431598 | -0.000002 |
| C5 | -0.232815 | 2.758933 | -0.000005 | H5 | 1.287547 | -2.903900 | -0.000007 |
| C6 | -1.517457 | 3.238388 | 0.000001 | H6 | 2.356673 | 1.927047 | 0.000006 |
| C7 | -2.402894 | 2.127067 | -0.000001 | C14 | 3.093612 | -0.070770 | 0.000001 |
| C8 | -3.113204 | -1.121963 | 0.000005 | H7 | 3.645898 | -2.145539 | -0.000003 |
| C9 | -2.771177 | -2.499519 | 0.000003 | F1 | 4.374662 | 0.349480 | 0.000004 |
| C10 | -1.402016 | -2.597413 | 0.000000 | H8 | -3.482367 | 2.151349 | 0.000000 |
| C11 | 2.091198 | 0.878194 | 0.000003 | H9 | -4.104482 | -0.693892 | 0.000008 |

**6,7-Difluorodipyrrolo[1,2-*a*:2′,1′-*c*]quinoxaline (1d) optimized by DFT calculation**

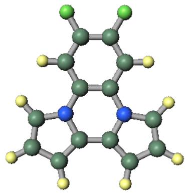

Total energy: -847.242917146 A.U.

Cartesian coordinates:

| | | | | | | | |
|---|---|---|---|---|---|---|---|
| C1 | 2.073781 | 0.718967 | 0.000000 | C12 | -1.584127 | 1.393318 | -0.000003 |
| N1 | 0.854722 | 1.381416 | -0.000002 | H1 | 0.284209 | -3.458908 | 0.000005 |
| C2 | -0.370218 | 0.703711 | -0.000002 | H2 | 2.938860 | -3.898603 | -0.000012 |
| C3 | -0.370121 | -0.703705 | 0.000000 | H3 | 2.937748 | 3.899123 | 0.000003 |
| N2 | 0.854955 | -1.381435 | 0.000001 | H4 | 0.283523 | 3.458886 | -0.000002 |
| C4 | 2.074079 | -0.718841 | 0.000001 | C13 | -2.774103 | 0.694409 | -0.000002 |
| C5 | 1.088862 | -2.742175 | 0.000003 | H5 | -1.622768 | 2.474672 | -0.000005 |
| C6 | 2.446550 | -2.937022 | -0.000007 | H6 | -1.622180 | -2.474506 | 0.000005 |
| C7 | 3.074794 | -1.663169 | 0.000000 | C14 | -2.774625 | -0.694873 | 0.000001 |
| C8 | 3.074320 | 1.663621 | 0.000004 | F1 | -3.938440 | 1.357114 | -0.000002 |
| C9 | 2.445888 | 2.937303 | 0.000002 | F2 | -3.938484 | -1.357643 | 0.000003 |
| C10 | 1.088159 | 2.742104 | 0.000000 | H7 | 4.134672 | -1.457109 | -0.000001 |
| C11 | -1.583854 | -1.393021 | 0.000002 | H8 | 4.134200 | 1.457583 | 0.000006 |

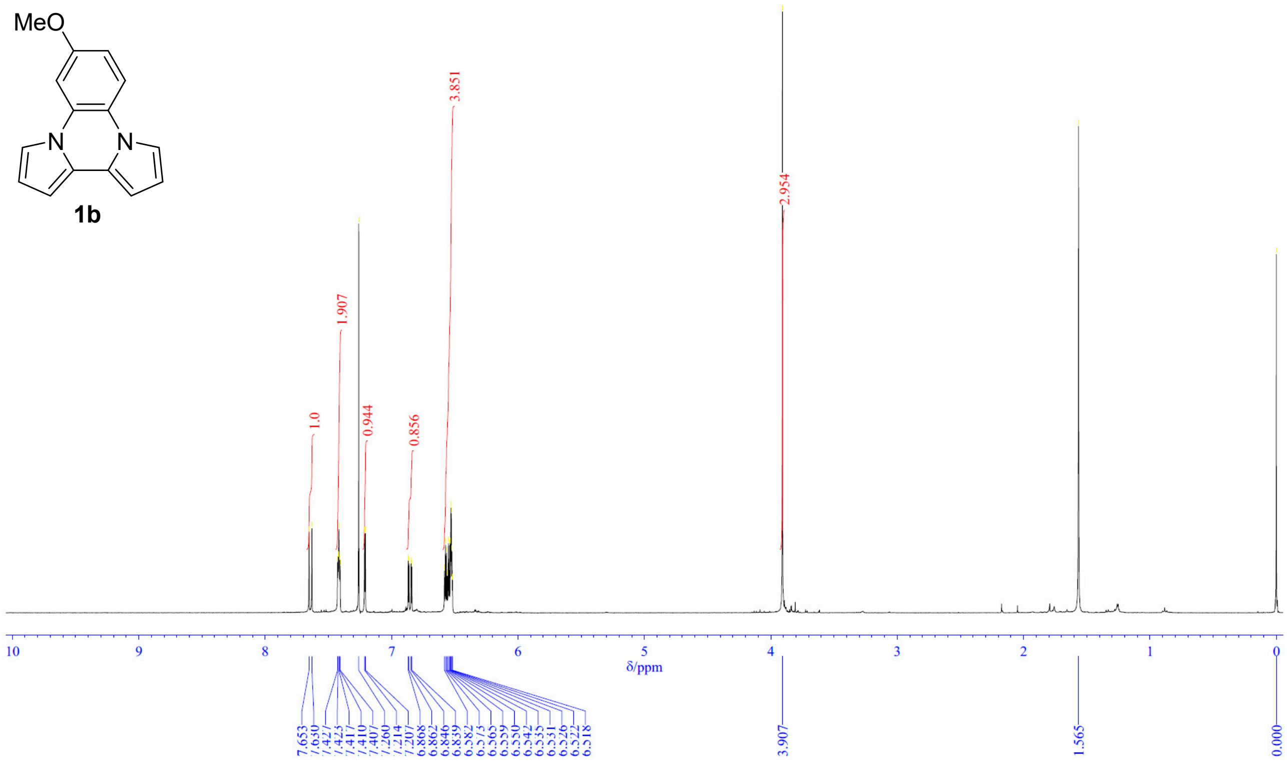
MeO
N
N
1b
1.0
1.907
0.944
0.856
3.851
2.954
δ/ppm
7.653
7.630
7.427
7.423
7.417
7.410
7.407
7.260
7.214
7.207
6.868
6.862
6.846
6.839
6.582
6.575
6.565
6.559
6.550
6.542
6.535
6.531
6.526
6.522
6.518
3.907
1.565
0.000

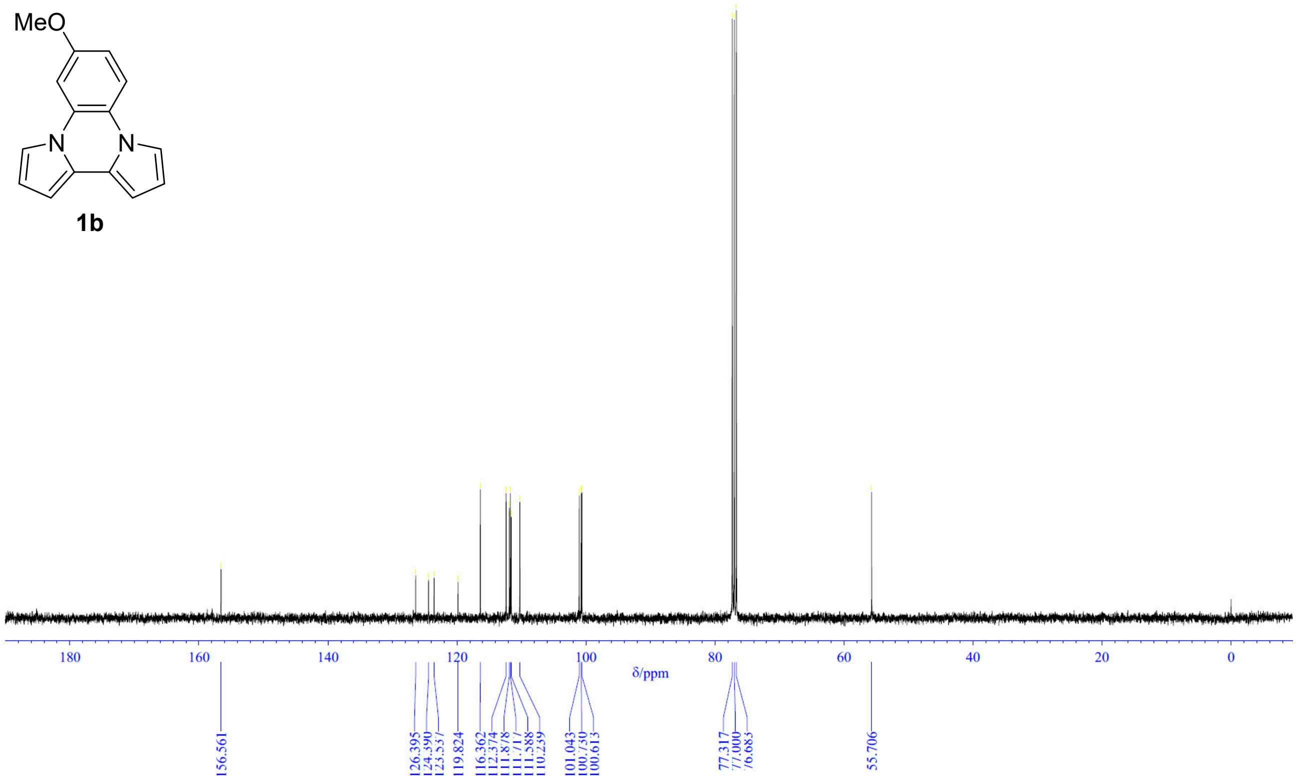
MeO
N
N
1b
δ/ppm
156.561
126.395
124.390
123.537
119.824
116.362
112.374
111.878
111.717
111.588
110.239
101.043
100.730
100.613
77.317
77.000
76.683
55.706

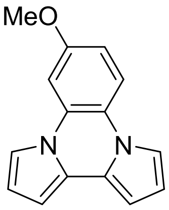


**1b**

\\EXACTIVE-W7\data\...\221028_3389_01 2022/10/28 11:52:12

RT: 0.00 - 1.05

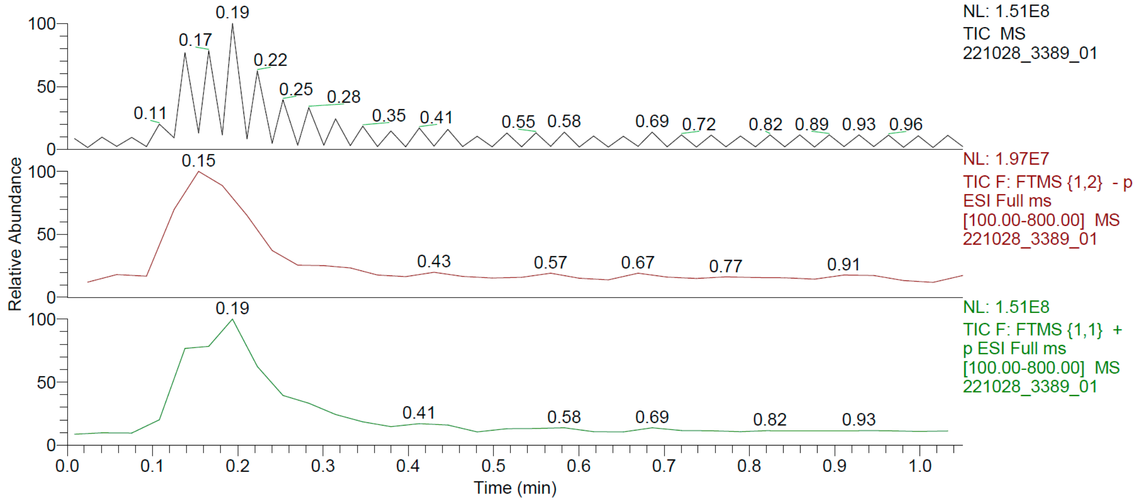


221028_3389_01 #12 RT: 0.18 AV: 1 NL: 1.41E6
T: FTMS {1,2} - p ESI Full ms [100.00-800.00]

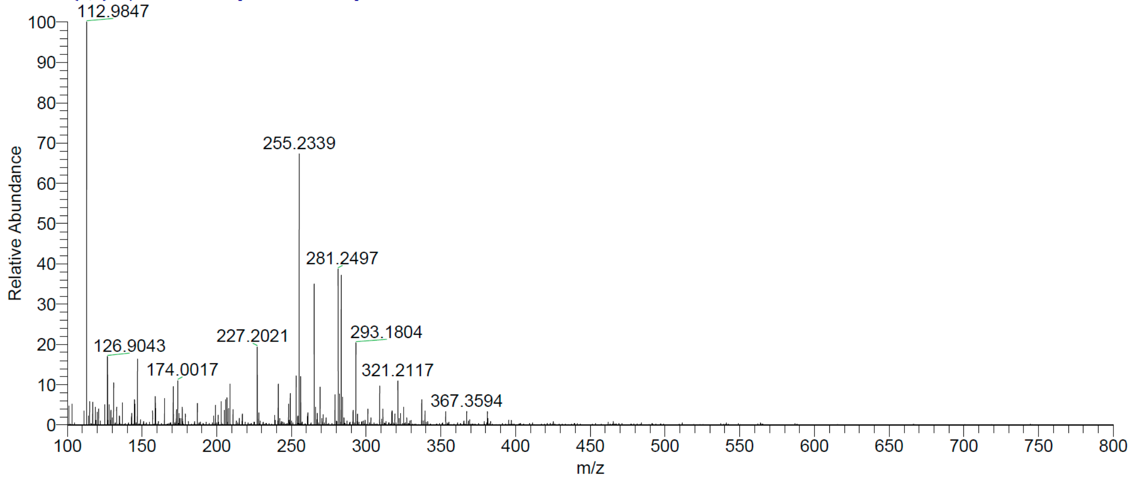


221028_3389_01 #10 RT: 0.15 AV: 1 NL: 1.56E4
T: FTMS {1,2} - p ESI Full ms [100.00-800.00]

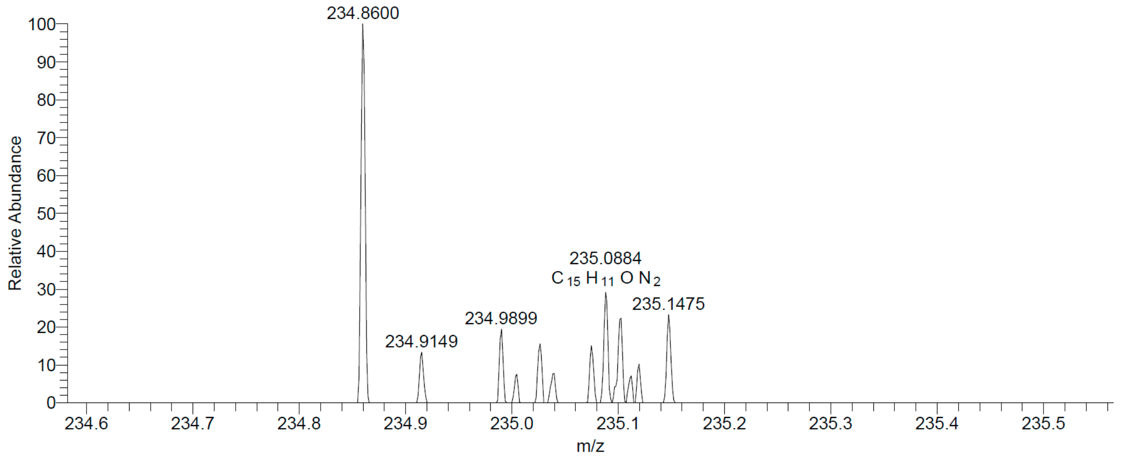

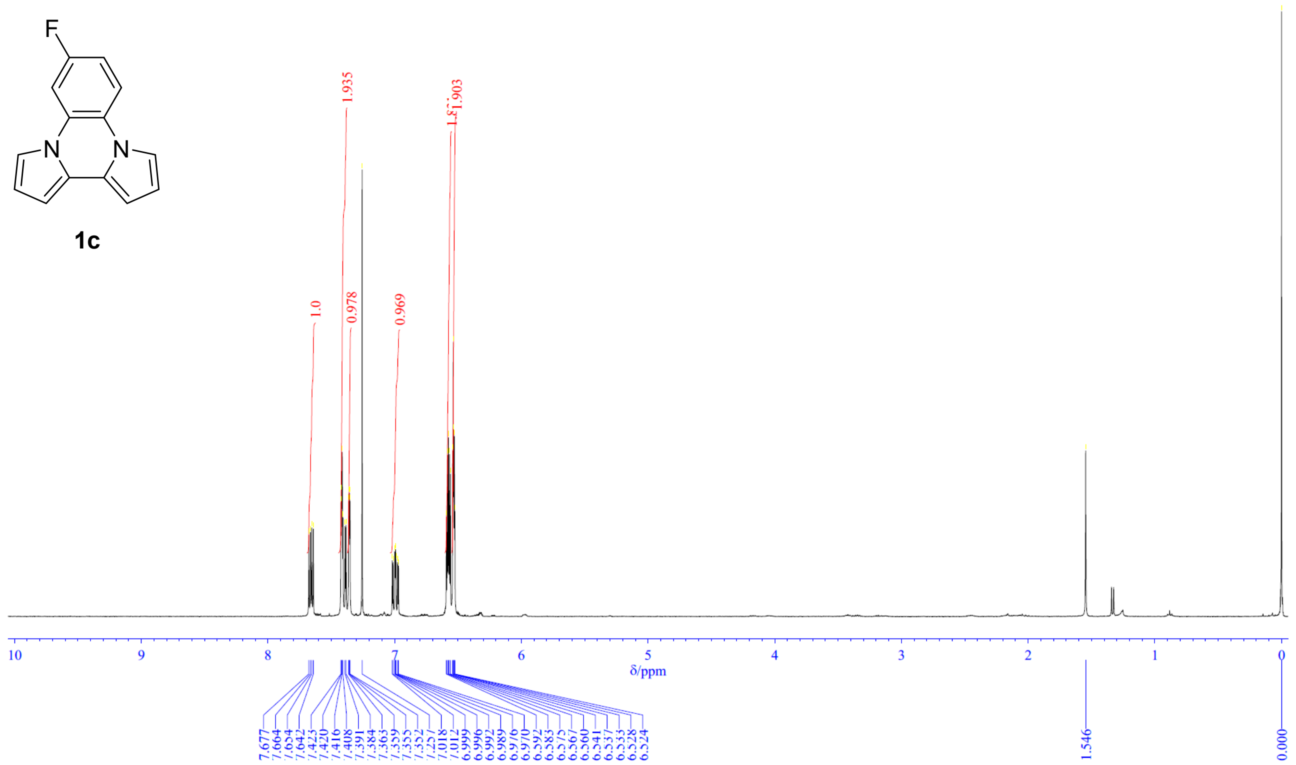

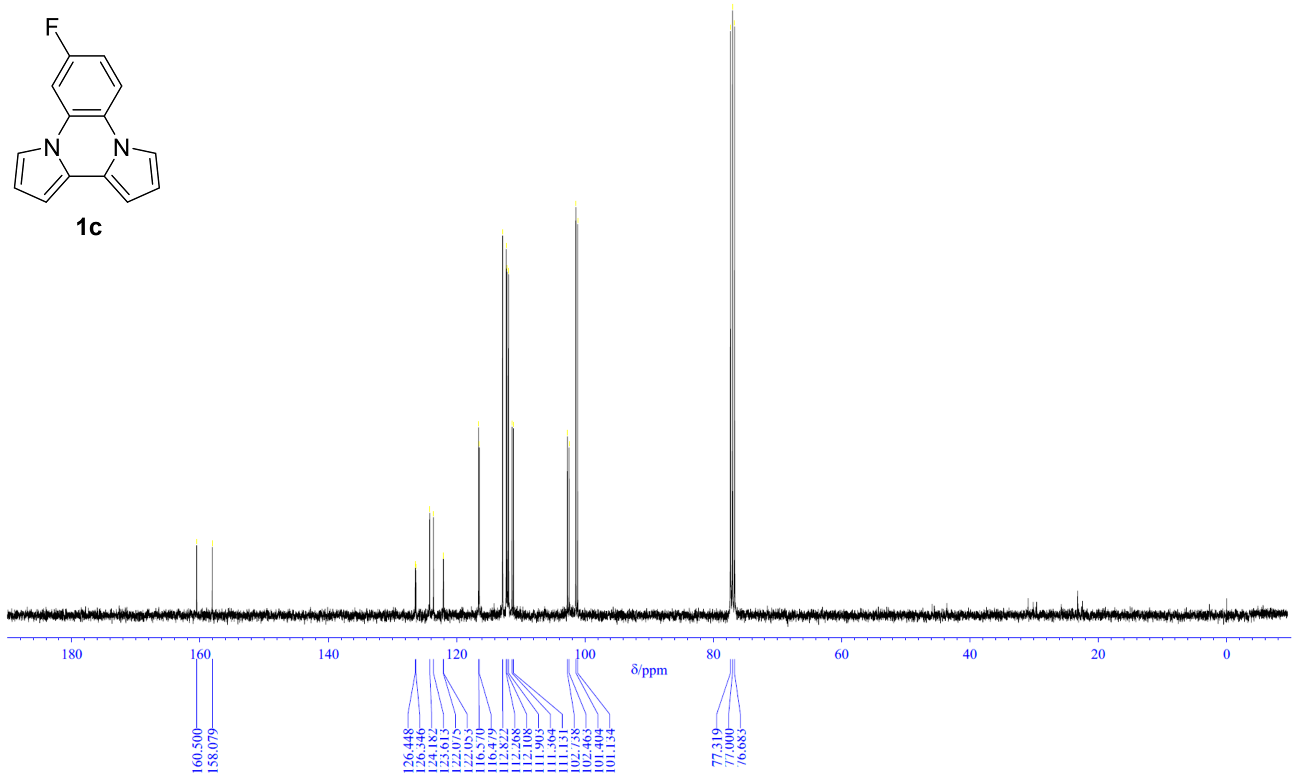

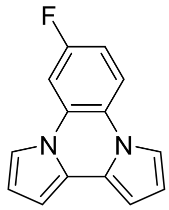


**1c**

\\EXACTIVE-W7\data\...\230106_3389_12 2023/01/06 12:40:42

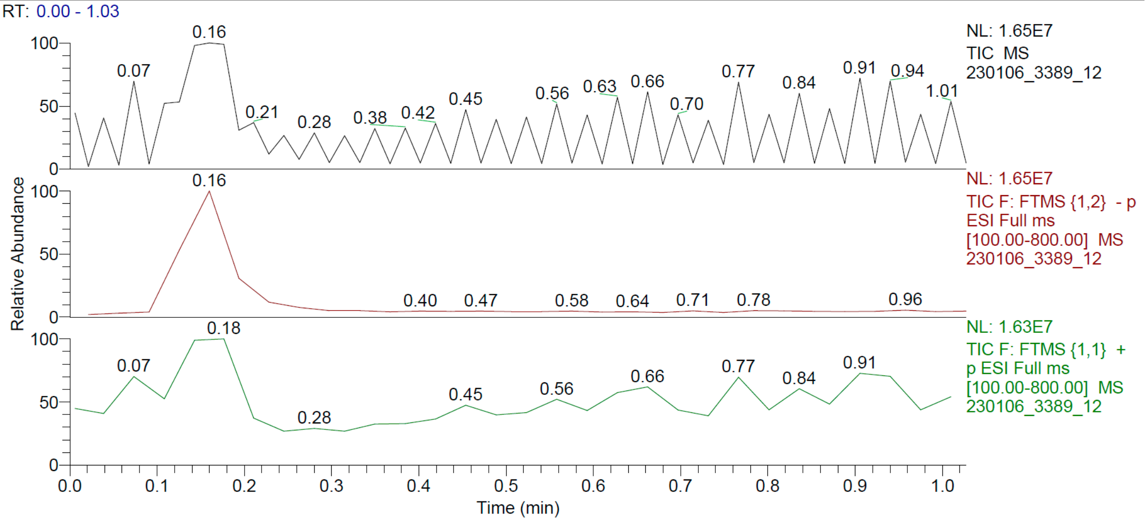


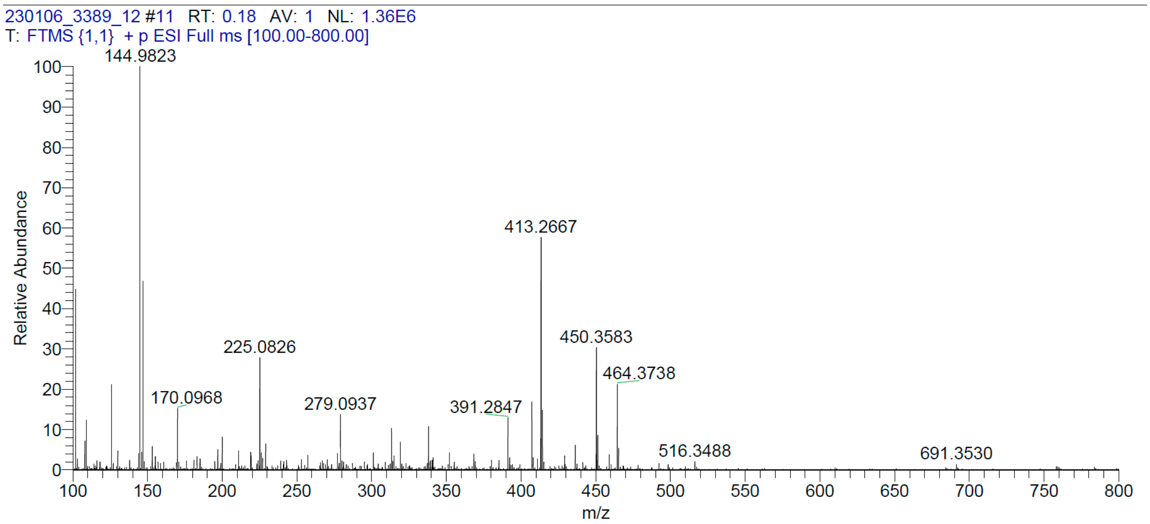


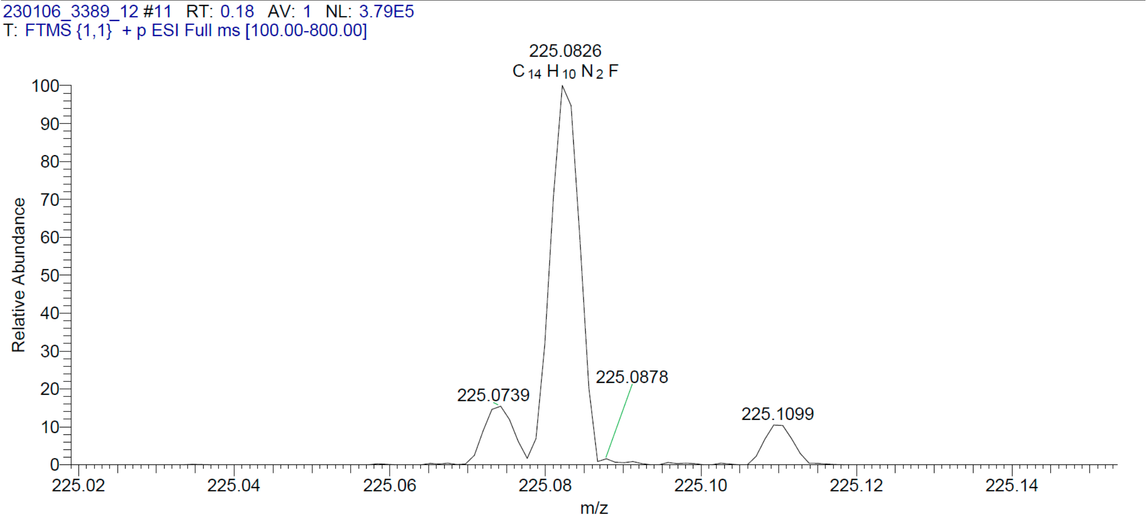

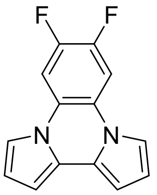
F
F
N
N

**1d**

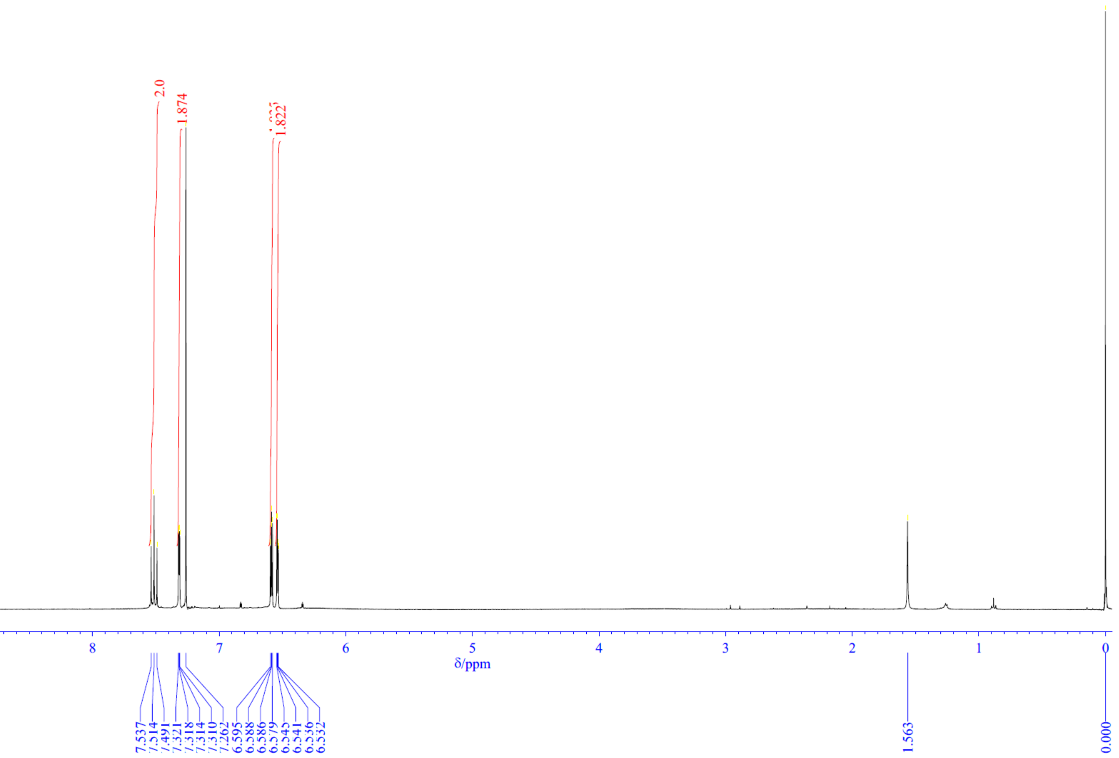
2.0
1.874
1.822
10
9
8
7
6
5
4
3
2
1
0
δ/ppm
7.537
7.514
7.491
7.321
7.318
7.314
7.310
7.262
6.595
6.588
6.586
6.579
6.545
6.541
6.536
6.532
1.563
0.000

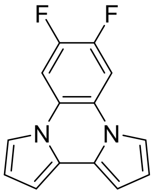
F
F
N
N

**1d**

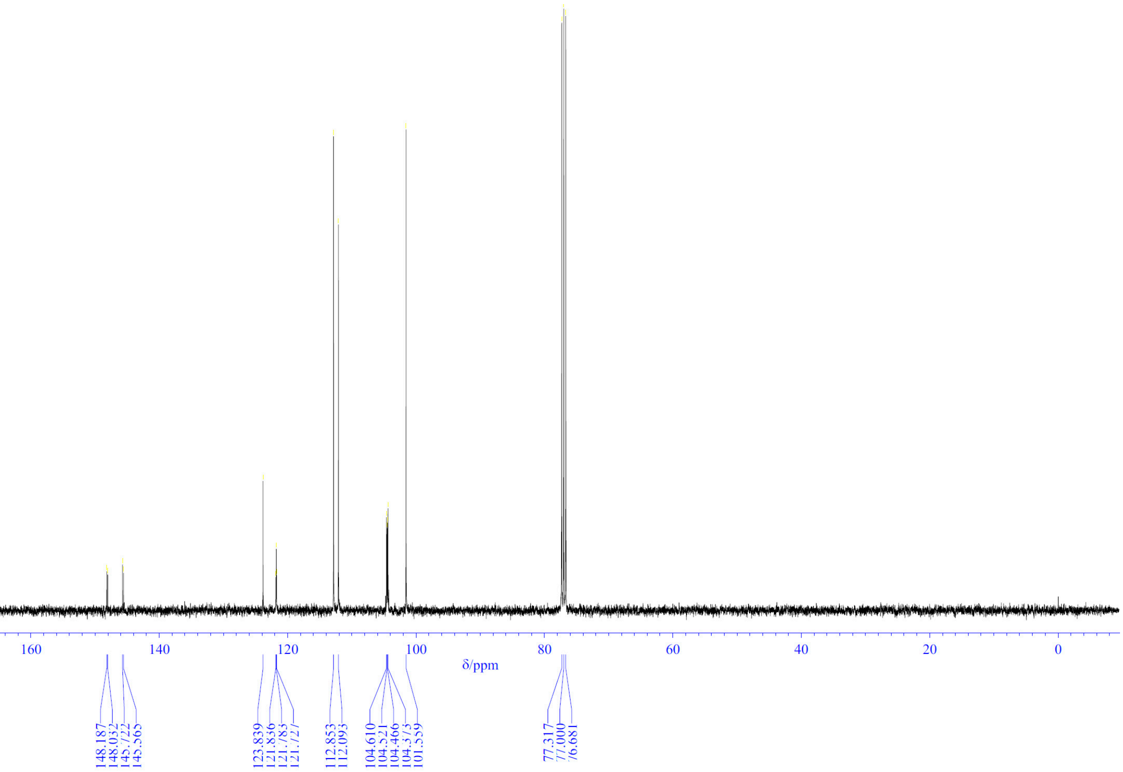
180
160
140
120
100
80
60
40
20
0
δ/ppm
148.187
148.032
145.722
145.565
123.839
121.856
121.785
121.727
112.853
112.093
104.610
104.521
104.466
104.375
101.559
77.317
77.000
76.681

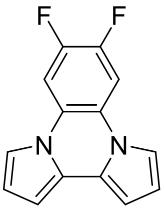


**1d**

\\EXACTIVE-W7\data\...\230106_3389_13 2023/01/06 12:44:20

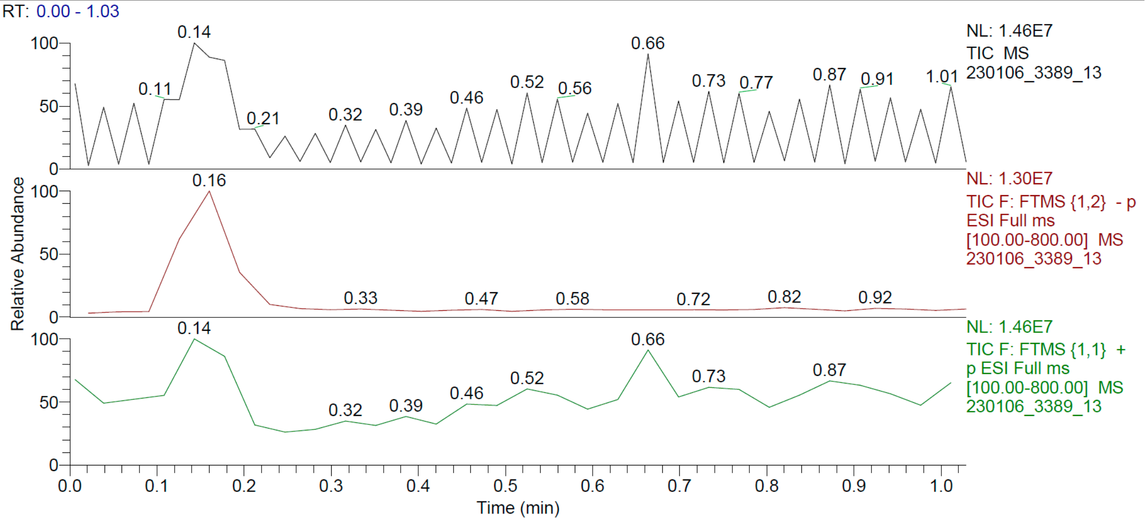


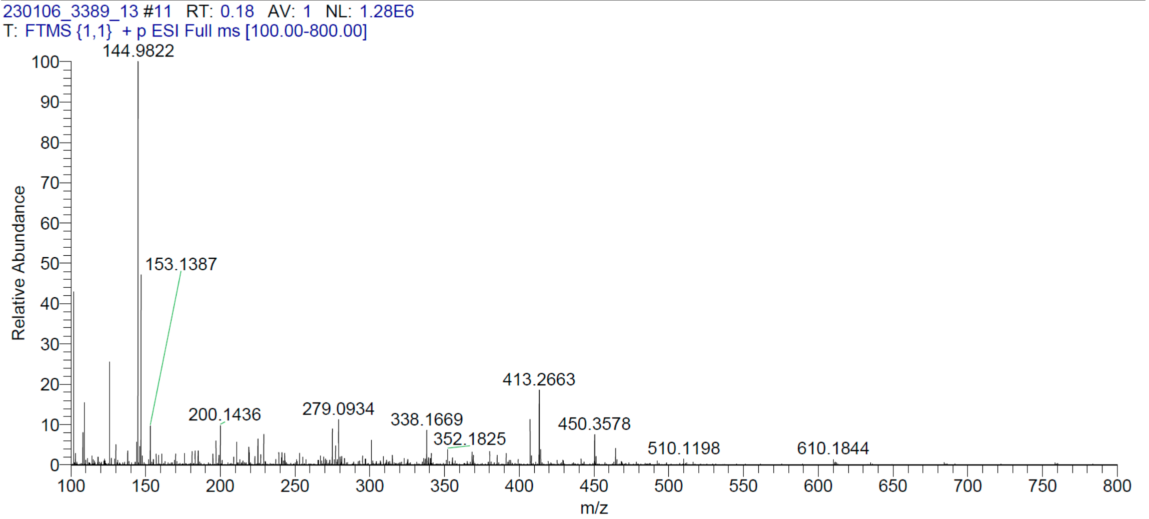


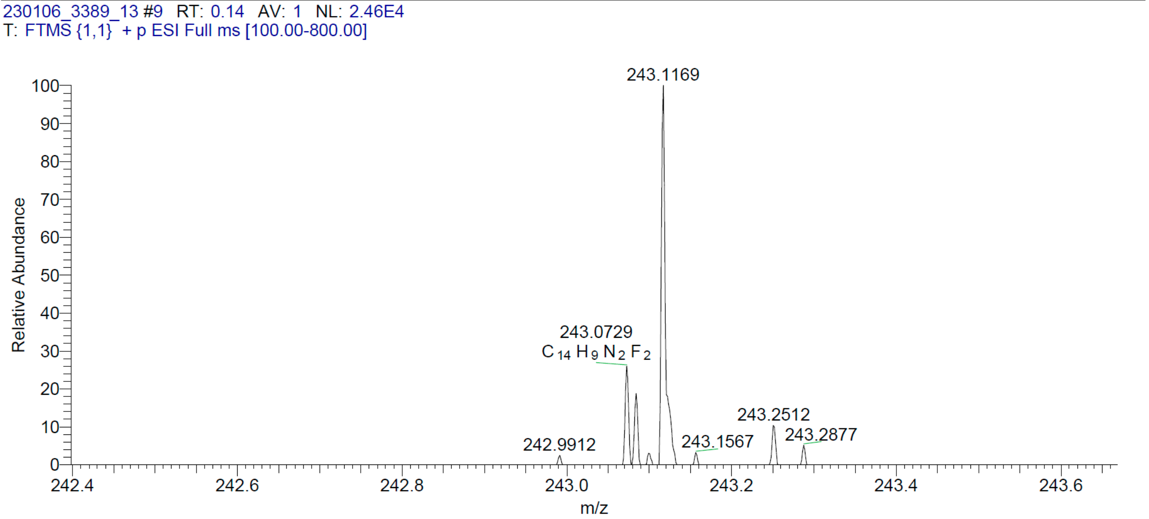

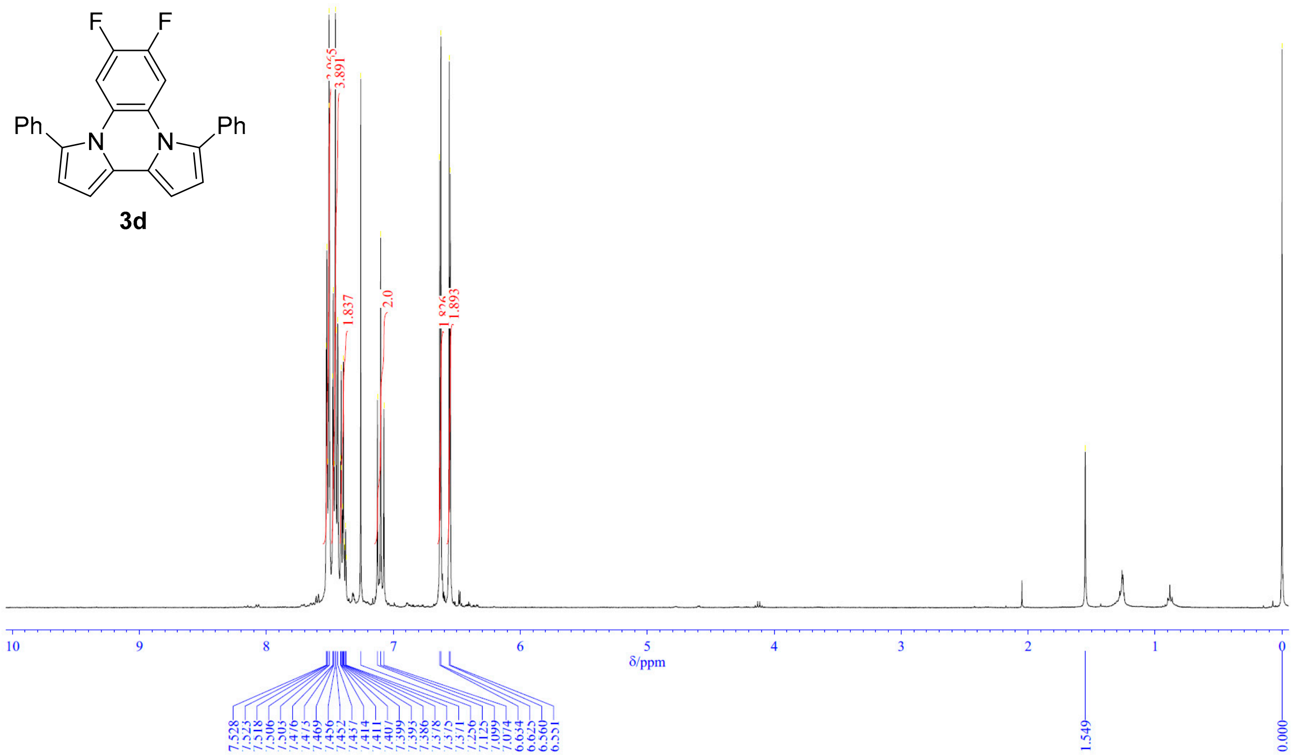
F
F
Ph
N
N
Ph
3d
3.891
1.837
2.0
1.893
10
9
8
7
6
5
4
3
2
1
0
δ/ppm
7.528
7.523
7.518
7.506
7.503
7.476
7.473
7.469
7.456
7.452
7.437
7.414
7.411
7.407
7.399
7.393
7.386
7.378
7.375
7.371
7.256
7.125
7.099
7.074
6.634
6.625
6.560
6.551
1.549
0.000

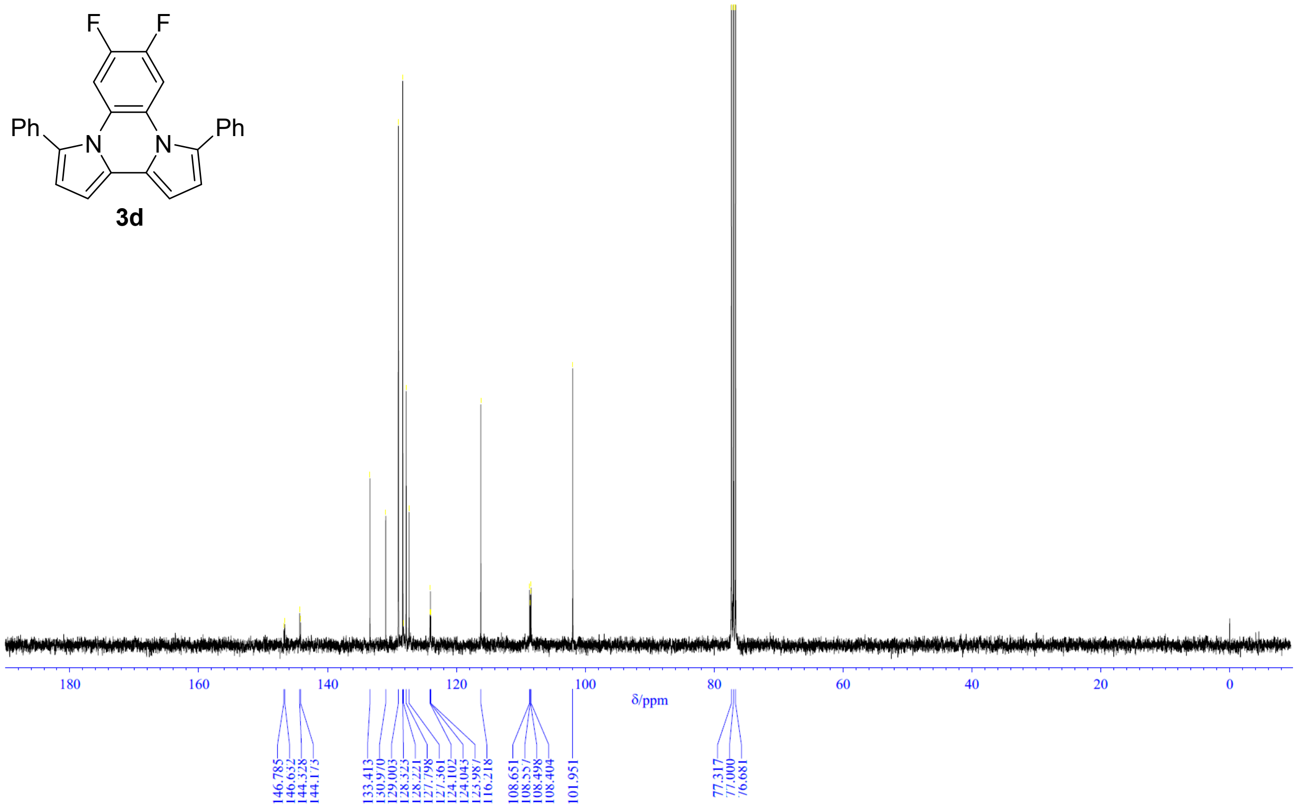
F
F
Ph
N
N
Ph
3d
180
160
140
120
100
80
60
40
20
0
δ/ppm
146.785
146.632
144.328
144.173
133.413
130.970
129.003
128.523
128.221
127.798
127.361
124.102
124.043
123.987
116.218
108.651
108.557
108.498
108.404
101.951
77.317
77.000
76.681

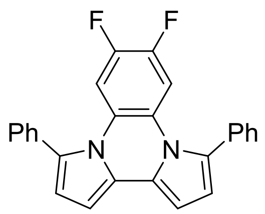


**3d**

\\EXACTIVE-W7\data\...\230106_3389_15 2023/01/06 12:51:43

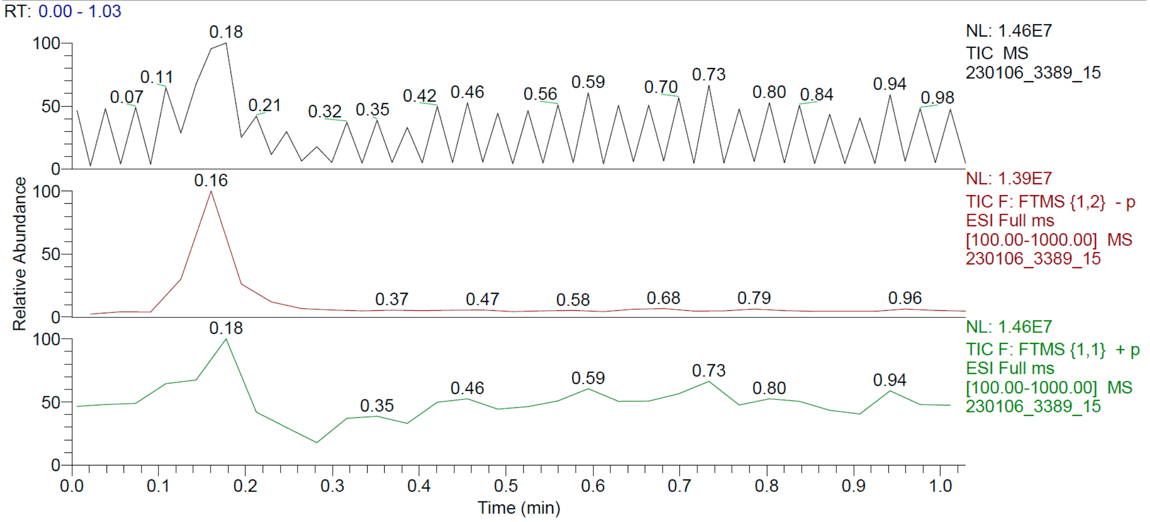


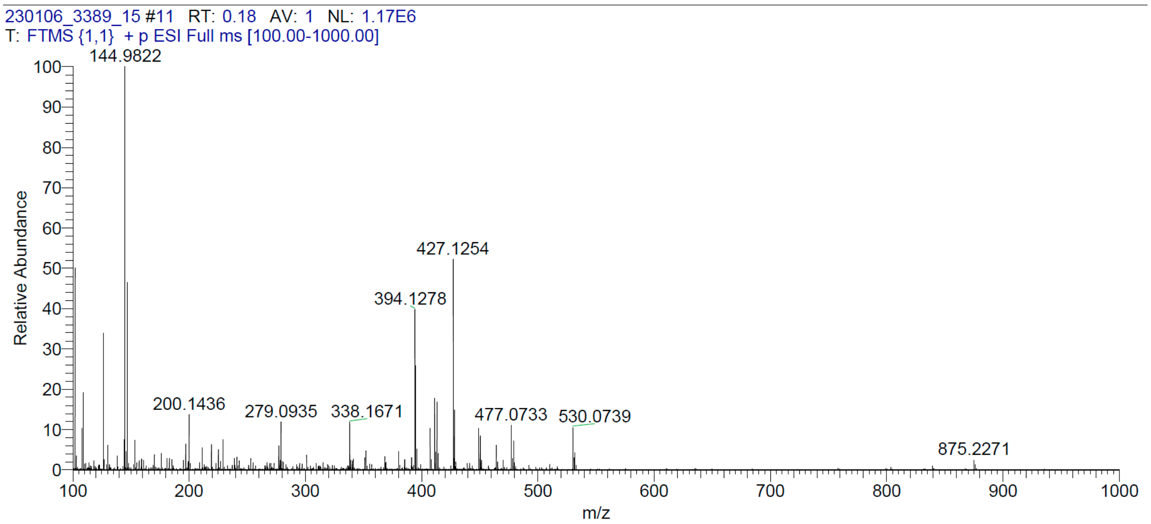


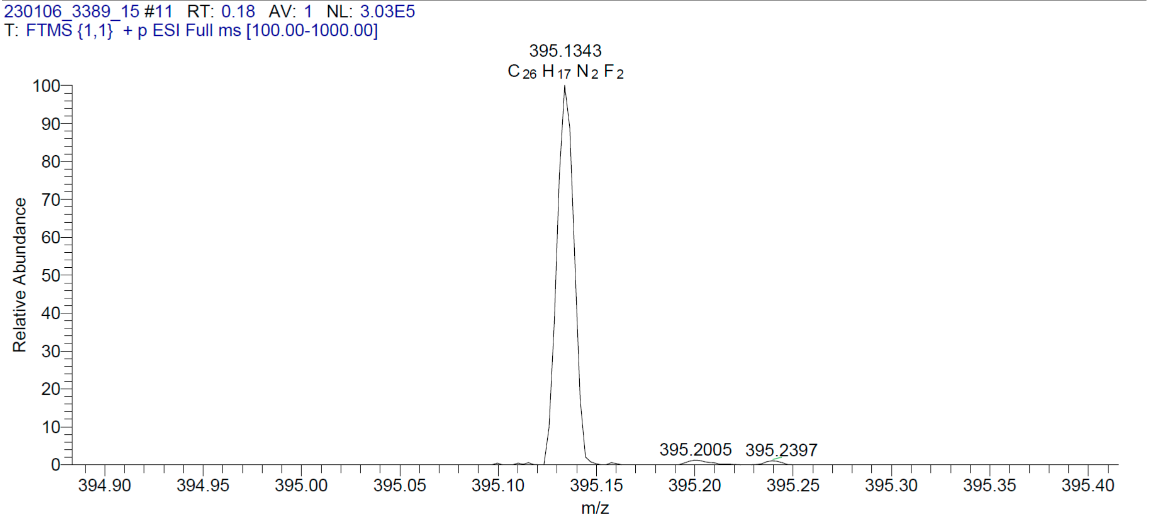

Supplement: Supplementary file 1 [file molecules-28-02896-s001.zip › molecules-2302023-supplementary.pdf]
